# Distinct roles of *PIK3CA* in the enrichment and maintenance of cancer stem cells in head and neck squamous cell carcinoma

**DOI:** 10.1002/1878-0261.12584

**Published:** 2019-10-26

**Authors:** Xi Chen, Yu Cao, Wafik Sedhom, Ling Lu, Yanqiu Liu, Haibo Wang, Masako Oka, Sophia Bornstein, Sherif Said, John Song, Shi‐Long Lu

**Affiliations:** ^1^ Department of Otolaryngology University of Colorado Anschutz Medical Campus Aurora CO USA; ^2^ Department of Surgical Oncology First Hospital of China Medical University Shengyang China; ^3^ Institute of Integrative Medicine Dalian Medical University China; ^4^ Department of Surgical Oncology Second Hospital of Dalian Medical University China; ^5^ Department of Radiation Oncology Cornell University New York NY USA; ^6^ Department of Pathology University of Colorado Anschutz Medical Campus Aurora CO USA; ^7^ Department of Dermatology University of Colorado Anschutz Medical Campus Aurora CO USA

**Keywords:** cancer stem cell, head and neck squamous cell carcinoma, *PIK3CA*, ponatinib, recurrence and metastasis

## Abstract

Recurrence and metastasis are the major causes of mortality in head and neck squamous cell carcinoma (HNSCC). It is suggested that cancer stem cells (CSCs) play pivotal roles in recurrence and metastasis. Thus, a greater understanding of the mechanisms of CSC regulation may provide opportunities to develop novel therapies for improving survival by controlling recurrence or metastasis. Here, we report that overexpression of the gene encoding the catalytic subunit of PI3K (*PIK3CA*), the most frequently amplified oncogene in HNSCC, promotes epithelial‐to‐mesenchymal transition and enriches the CSC population. However, *PIK3CA* is not required to maintain these traits and inhibition of the phosphatidylinositol 3‐kinase (PI3K) signaling pathway paradoxically promotes CSC population. Molecular analysis revealed that overexpression of *PIK3CA* activates multiple receptor tyrosine kinases (RTKs), in which ephrin receptors (Ephs), tropomyosin receptor kinases (TRK) and mast/stem cell growth factor receptor (c‐Kit) contribute to maintain CSC population. Accordingly, simultaneous inhibition of these RTKs using a multi‐kinase inhibitor ponatinib has a superior effect at eliminating the CSC population and reduces metastasis of *PIK3CA*‐overexpressing HNSCC cells. Our result suggests that co‐targeting of Ephs, TRKs and the c‐Kit pathway may be effective at eliminating the PI3K‐independent CSC population, thereby providing potential targets for future development of a novel anti‐CSC therapeutic approach for HNSCC patients, particularly for patients with *PIK3CA* amplification.

## Graphical abstract

We report that overexpression of the gene encoding the catalytic subunit of PI3K (PIK3CA) promotes epithelial‐to‐mesenchymal transition and enriches the cancer stem cell (CSC) population. However, PIK3CA is not required to maintain these traits. Molecular analysis revealed that PIK3CA overexpression activates multiple receptor tyrosine kinases (RTK), in which tropomyosin receptor kinases, ephrin receptors, and mast/stem cell growth factor receptor (c‐Kit) contribute to maintain CSCs. Accordingly, co‐inhibition of these RTKs using a multi‐kinase inhibitor ponatinib eliminates CSCs and reduces metastasis of PIK3CA‐overexpressing head and neck squamous cell carcinoma cells.

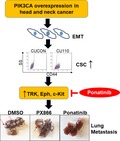

**Abbreviations**
4NQO: 4‐nitroquinoline 1‐oxide
c‐Kit: mast/stem cell growth factor receptor
CSCs: cancer stem cells
EMT: epithelial‐to‐mesenchymal transition
Ephs: ephrin receptors
GEMM: genetically engineered mouse model
H&E: hematoxylin and eosin
HNSCC: head and neck squamous cell carcinoma
HPV: human papilloma virus
IF: immunofluorescence
PI3K: phosphatidylinositol 3‐kinase
*PIK3CA*: the gene encoding the catalytic subunit of PI3K
*PIK3R1*: the gene encoding the regulatory subunit of PI3K
RTK: receptor tyrosine kinases
sh: short hairpin
SP: side population
TRK: tropomyosin receptor kinases

## 1 Introduction

Head and neck squamous cell carcinoma (HNSCC) refers to cancer derived from the squamous epithelium along the head and neck region, including nasal cavity, oral cavity and tongue, pharynx (nasal pharynx, oropharynx, hypopharynx) and larynx. It affects 650 000 people and claims 350 000 lives worldwide annually (Torre *et al.*, 2015). In the USA alone, there are 65 000 new cases of HNSCC and 15 000 deaths every year (Siegel *et al.*, 2017). Tobacco, alcohol and human papilloma virus (HPV) infection are known risk factors for HNSCC (Haddad and Shin, 2008; Maxwell *et al.*, 2016). HNSCC is still a deadly cancer with an average of 50% overall 5‐year survival rate (Pulte and Brenner, 2010). The main reasons for the poor prognosis of HNSCC patients are loco‐regional invasions, treatment resistance, recurrence and metastasis (Leemans *et al.*, 2011). On initial presentation, ~ 10% of HNSCC cases show metastases and the survival rate for these patients is less than 1 year (Argiris *et al.*, 2008). Additionally, ~ 30–40% of post‐treated HNSCC patients develop recurrence or metastasis (Argiris *et al.*, 2008). Thus, therapies controlling HNSCC recurrence and/or metastasis are pivotal to improve poor survival of HNSCC patients.

The phosphatidylinositol 3‐kinase (PI3K) signaling pathway is the most frequently altered oncogenic pathway in HNSCC (Du *et al.*, 2012; Iglesias‐Bartolome *et al.*, 2013; Lui *et al.*, 2013; Pickering *et al.*, 2013; The Cancer Genome Atlas, 2015; Vander Broek *et al.*, 2015). The PI3K is composed of a heterodimer of a p110 catalytic subunit and a p85 regulatory subunit (Thorpe *et al.*, 2015). Recent whole‐exome sequencing of HNSCC samples has identified mutations (10%) and/or amplification (40%) of the gene encoding the catalytic subunit of PI3K (*PIK3CA*), the gene encoding for p110α subunit of PI3K, making it the most commonly altered oncogene in human HNSCC patients (Iglesias‐Bartolome *et al.*, 2013; Lui *et al.*, 2013; Pickering *et al.*, 2013; The Cancer Genome Atlas, 2013). To better understand the role of *PIK3CA* in the development and progression of HNSCC *in vivo*, we previously overexpressed *PIK3CA* in the head and neck epithelium using an inducible head‐and‐neck‐specific genetically engineered mouse model (*PIK3CA*‐GEMM) (Du *et al.*, 2016). We showed that although overexpression of *PIK3CA* was not sufficient to initiate tumorigenesis, it markedly accelerated HNSCC progression, manifested as poorly differentiated and metastatic tumors. These tumors exhibited a phenotype of epithelial‐to‐mesenchymal transition (EMT) and increased gene expression related to EMT and cancer stem cells (CSCs). These data suggested that these two factors might act together to drive tumor invasion and metastasis, thereby promoting HNSCC progression.

The EMT is a process by which epithelial cells lose their cell polarity and cell‐to‐cell adhesion, and gain an elongated, fibroblast‐like morphology. EMT is a fundamental event in developmental morphogenesis (Nieto, 2011). Cancer cells can hijack the EMT program to gain several functions related to cancer progression, such as enhanced migration and invasion, resistance to anoikis and chemotherapies, generation of immune‐suppressive environments, and gain of CSC properties, or ‘cancer stemness’ (Brabletz *et al.*, 2018; De Craene and Berx, 2013; Nieto, 2013). CSCs are a small population of stem‐like cancer cells that share the properties of self‐renewal and multipotency with stem cells from normal tissue (Gupta *et al.*, 2019; Nassar and Blanpain, 2016; Nguyen *et al.*, 2012)*.* Since CSCs possess self‐renewal and tumorigenic properties, and are in general quiescent and require less nutrients, it is believed that they are more suitable to survive in a harsh environment, resistant to chemo‐radiation therapies, and can be ‘seeds’ for tumor formation primarily (tumor initiation), secondarily (recurrence) or distantly (metastasis) (Batlle and Clevers, 2017; Gupta *et al.*, 2019). Thus, targeting CSCs has recently become a promising target for treatment of primary cancer, treatment resistance, recurrence and metastasis (Brown *et al.*, 2017; Chen *et al.*, 2017; Kerk *et al.*, 2017; Saygin *et al.*, 2019).

To understand the role of EMT and CSC traits in HNSCC progression, we further investigated these two processes using both murine HNSCC cell‐derived tumors from the *PIK3CA*‐GEMM and human HNSCC cell lines. Our data revealed that *PIK3CA* overexpression promotes EMT and enriches CSCs in both murine and human HNSCC cell lines. Surprisingly, inhibition of *PIK3CA* or key components of the PI3K pathway did not affect the CSC pool. To reveal the molecular mechanism of resistance to PI3K inhibition, we performed a receptor tyrosine kinase (RTK) array and found that multiple RTKs were activated in the *PIK3CA*‐overexpressing murine HNSCC cells. Pharmaceutical inhibitor screening showed that targeting Ephrin receptors (Ephs), tropomyosin receptor kinases (TRKs) or mast/stem cell growth factor receptor (c‐Kit) reduced the CSC population. A multi‐RTK inhibitor named ponatinib, which targets Ephs, TRKs and c‐Kit, completely eliminated CSC population *in vitro* and significantly reduced lung metastasis *in vivo*, suggesting that targeting these RTKs by ponatinib may be an effective therapeutic strategy targeting CSCs in HNSCC with *PIK3CA* amplification.

## 2 Materials and methods

### 2.1 Cell culture

Cells were cultured from either 4‐nitroquinoline 1‐oxide (4NQO)‐induced control tongue SCCs (CUCONs) or 4NQO‐induced *PIK3CA*‐GEMM tongue SCCs (CU110s). Tumor tissues were minced with a blade and digested in 0.35% collagenase (Gibco, Carlsbad, CA, USA) followed by two rounds of 1% trypsin digestion at 37 °C. Single cells were obtained through a cell strainer (70 µm nylon; BD Biosciences, Franklin Lakes, NJ, USA) and plated at 1 × 10^5^ per mL in 10‐cm dishes. The authentication of the cultured cells was validated by the transgene‐specific genotyping PCR. The CU110 and CUCON cells were maintained in Dulbecco’s modified Eagle’s medium (DMEM; Corning Cellgro, Corning, NY, USA) supplemented with 10% FBS (Sigma‐Aldrich, St. Louis, MO, USA), streptomycin (100 mg·mL^−1^) and penicillin (100 IU·mL^−1^) in a 5% CO_2_ incubator (37 °C).

UMSCC cell lines were obtained from the University of Michigan and VU cell lines from Vrije University in the Netherlands under MTA agreements. Human HNSCC cell lines UM‐SCC‐1, UM‐SCC‐2, UM‐SCC‐47, VU1131, VU1365, Fadu, LNM1, Tu167, HN6, CAL27, M4C and M4E were obtained and cultured as recommended by protocols provided by University of Michigan, Vrije University or ATCC. These cells were cultured in DMEM (Corning Cellgro) supplemented with 10% FBS (Sigma‐Aldrich), streptomycin (100 mg·mL^−1^) and penicillin (100 IU·mL^−1^) in a 5% CO_2_ incubator (37 °C).

### 2.2 Cell proliferation assay

For the cell proliferation assays, cells were seeded in 12‐well plates at a density of 5 × 10^4^ cells per well in triplicate. Cells were then counted with a Vi‐CELL cell counter (Beckman Coulter, Brea, CA, USA) at different time points. Briefly, cells in each well were harvested by treating with trypsin. An 800‐μL diluted cell suspension was used for the cell viability analysis with a Vi‐CELL counter. We also used Cell Counting Kit‐8 (Dojindo, Kumamoto, Japan) to quantify cell proliferation and viability according to the manufacturer’s manual. Briefly, 6 × 10^3^ cells were seeded into each well of 96‐well plate. After different time points, 100 µL of Cell Counting Kit‐8 reagent was added to each well and incubated at 37 °C for 1.5 h. The plate was then read by plate reader (BioTek Synergy II, Winooski, VT, USA) at 450 nm.

### 2.3 Stable knocking down using lentiviral‐based shRNA

The following lentiviral‐based vectors containing short hairpin (sh)‐RNA were purchased from Sigma‐Aldrich (Table S1). For lentiviral transfection, 2.2 × 10^4^ CU110 cells (or 3.5 × 10^4^ cells for Fadu/UMSCC47) were plated in each well of a 6‐well plate and virus‐containing media with 8 mg·mL^−1^ polybrene (Millipore, Burlington, MA, USA) was added on the second day. Forty‐eight hours later, complete media with puromycin (10 mg·mL^−1^ for CU110 cells and 2 mg·mL^−1^ for Fadu/UMSCC47 cells) was added. Cells were cultured with puromycin for 1 week to eliminate non‐transduced cells. Knockdown efficiency was determined by quantitative real‐time PCR and western blotting analysis.

### 2.4 HNSCC sphere formation assay

Either murine or human HNSCC cells or cells pretreated with inhibitors were suspended at a density of 4.0 × 10^5^ cells mL^–1^ in serum‐free DMEM (Corning Cellgro) medium and seeded into 96‐well ultralow attachment plates (Corning Incorporated, Corning, NY, USA) as 150 µL per well. Cells were cultured in a 5% CO_2_ incubator (37 °C). After 48 h, spheres (Sphe) (≥ 1.5 μm) were counted as an HNSCC Sphe‐forming unit using a bright‐field microscope (Leica, Wetzlar, Germany). The data were presented as the average of three independent experiments. Spheroids were cultured in the serum‐free DMEM medium for 2–12 weeks before mRNA extraction, protein isolation, histology or *in vivo* experiments.

### 2.5 FACS analysis

All antibodies used for FACS analysis were purchased from eBioscience (San Diego, CA, USA) unless specified otherwise. In brief, murine or human HNSCC cells or cells treated with inhibitors were harvested and washed twice in PBS buffer, and suspended in PBS with 1% serum at a density of 1.0 × 10^6 ^cells/100 µL. Cells were then stained with fluorochrome‐conjugated monoclonal antibodies for mouse CD24 (17‐0242‐82), mouse/human CD44 (48‐0441‐82), for 1 h on ice. After washing twice with ice‐cold PBS, cells were re‐suspended in 400 µL ice‐cold PBS with 1% serum. Propidium iodide (Sigma‐Aldrich) was added (1 µg·mL^−1^) to exclude dead cells for the analysis. A minimum of 50 000 events were recorded for all samples. All FACS analyses were performed on a Gallios (Beckman Coulter) and the data were analyzed using Kaluza (Beckman Coulter).

The assessment of ALDH1 activity was conducted using ALDEFLUOR assay (StemCell Technologies, Durham, NC, USA). The procedure followed the manufacturer’s manual. In brief, the single cell suspension was washed twice in PBS buffer and then suspended in ALDEFLUOR assay buffer at a density of 0.8 × 10^6^ cells per mL. Activated ALDH substrate BAAA was added as 5 µL·mL^−1^ of cell suspension and then 500 µL was transferred to a tube containing 5 μL of 1.5 mm DEAB, a specific ALDH inhibitor. Cells were incubated at 37 °C for 45 min. After washing twice with ice‐cold PBS, cells were re‐suspended in 400 µL ice‐cold ALDEFLUOR assay buffer. Propidium iodide (Sigma‐Aldrich) was added (1 µg·mL^−1^) to exclude dead cells for the analysis. A minimum of 50 000 events were recorded for all samples. All flow cytometric analyses were performed on a Gallios (Beckman Coulter) and the data were analyzed using Kaluza (Beckman Coulter).

For the side population (SP) analysis, cells were suspended in the complete DMEM medium as 1.0 × 10^6^ cells mL^–1^. Hoechst 33342 (Sigma‐Aldrich) was then added at a final concentration of 5 µg·mL^−1^ for CU110 and CUCON cells and 1.5 µg·mL^−1^ for Fadu and UMSCC47 cells, and the samples were incubated for 90 min at 37 °C. After staining, cells were washed twice with ice‐cold PBS and re‐suspended in 400 µL of ice‐cold PBS with 1% serum for FACS analysis using a MoFlo XDP70 analyzer (Beckman Coulter). Immediately before the analysis, propidium iodide (Sigma‐Aldrich) was added (1 µg·mL^−1^) to exclude the dead cells. For the control reactions, CU110 or CUCON cells were incubated with 1 µmol of verapamil (or 0.7 µmol for Fadu and UMSCC47) for 30 min at 37 °C prior to the staining by Hoechst 33342.

### 2.6 Quantitative real‐time PCR

Total RNAs were extracted using RNeasy kits (Qiagen, Hilden, Germany). cDNAs for differential gene expression analysis were synthesized using a high‐capacity cDNA reverse transcription kit (Applied Biosystems, Foster City, CA, USA) according to the manufacturer’s instructions. Quantitative RT‐PCRs were carried out in triplicate using iTaq Universal SYBR Green Supermix (Bio‐Rad, Hercules, CA, USA) and were run on CFX Connect Real‐Time PCR Detection System (Bio‐Rad Laboratories, Inc.). Gene expression levels were normalized to GAPDH and quantified using a comparative CT method. The primer sequences used for gene expression analysis are listed in Table S2.

### 2.7 Protein analysis

Protein was extracted using the methods described previously (Du *et al.*, 2016). For western blot analysis, antibodies against p110α (4249s), total AKT (9272s), AKT1 (2938s), AKT2 (2964s), pAKT^Ser473^ (3787s), E‐cadherin (3195s), vimentin (5741s), SOX2 (3579s) and GAPDH (5174s) were purchased from Cell Signaling (Danvers, MA, USA,). Antibodies against p85α (ab71925) and ki67 (ab16667) were obtained from Abcam. Antibody against CD44 (550536) was obtained from BD Biosciences Pharmingen (San Jose, CA, USA). For the western blot, an equal amount of total protein (30–35 µg) was loaded onto an SDS/PAGE gel. After transferring to poly(vinylidene difluoride) membrane, the blot was incubated with antibodies. Blot images were captured by either a ChemiDoc MP system (Bio‐Rad Laboratories Inc.) or X‐ray film (Kodak, Rochester, NY, USA). Densitometric analysis was done with image j software (Bethesda, MD, USA).

### 2.8 Immunostainings

For the immunofluorescence (IF) staining, cells grown on the Lab‐Tek II chamber slide system (Thermo Fisher Scientific Inc.) or frozen tissue sections of Sphes were fixed in ice‐cold acetone for 10 min at 4 °C. Slides were washed in PBS and blocked with 5% goat serum for 1 h at room temperature and then incubated with primary antibodies for 30 min. After washing with PBS three times, slides were incubated with either Alexa 488‐conjugated (green; Invitrogen, Carlsbad, CA, USA) or Alexa 594‐conjugated (red; Invitrogen) secondary antibodies for 1 h. After a final wash with PBS, coverslips were mounted with EverBrite Mounting Medium with 4′,6‐diamidino‐2‐phenylindole (DAPI; Biotium Inc., Fremont, CA, USA) and examined using confocal microscopy. Double IF staining was performed as previously described (Bornstein *et al.*, 2009). Incubation with primary antibodies was as follows: E‐cadherin (Cell Signaling, #3195) and vimentin (BD Pharmingen, San Jose, CA, USA, RV202). We also performed immunohistochemistry (IHC) on the 4NQO‐induced tumors from either *PIK3CA* or control mice according to the protocol we described previously. The antibodies for IHC are CD44 (BD Pharmingen, #550538), BMI1 (Cell Signaling, #6964) and SOX2 (Cell Signaling, #14962). Slides were examined with a Leica microscope, and images were taken using the q capture pro software (Q imaging, British Columbia, Canada).

### 2.9 Receptor tyrosine kinase (RTK) antibody array

The PathScan RTK Signaling Antibody Array Kit was purchased from Cell Signaling Technologies, and the procedure was performed according to the instructions of the manufacturer. Briefly, protein lysates were prepared using RTK array lysis buffer; protein lysates were then quantified and diluted to the same concentration before loading to the array chamber. Detection antibody cocktail was then added to the chamber and incubated overnight at 4 ºC. After incubation, the chamber was washed . using RTK array wash buffer and processed with HRP‐linked Streptavidin solution. Chamber slide was documented . using the Bio‐Rad ChemiDoc XRD imaging system and dot density was quantified by using photoshop (Adobe, San Jose, CA, USA).

### 2.10 Inhibitor treatment

Information about all inhibitors used is listed in Table S3. All inhibitors were prepared in DMSO as 10 or 20 mm stock. The effect of the various treatments on cell viability was evaluated by two methods. The first method used the Vi‐CELL cell counter (Beckman Coulter). Briefly, cells were plated in 24‐well plates at a density of 9.4 × 10^4^ cells per well in triplicate. Cells were then treated with either vehicle (DMSO) or inhibitors. After 48 h, cells were trypsinized and cell viability was measured with a Vi‐Cell cell counter (Beckman Coulter). The percentage of viable cells was defined as the ratio of cell number in the inhibitor‐treated group to that of the DMSO‐treated group. IC_50_ was calculated using graphpad prism software (San Diego, CA, USA). We also used Cell Counting Kit‐8 (Dojindo) to quantify the cell viability according to the instruction manual.

### 2.11 *In vivo* tumorigenicity study

All animal experiments were performed in accordance with protocols approved by the Institutional Animal Cancer and Use Committees of University of Colorado Anschutz Medical Campus. For *in vivo* tumorigenicity study of HNSCC Sphes, monolayer (mono)‐growing or Sphe‐growing CU110 cells were injected subcutaneously in the left and right flanks of C57BL/6 mice at 10 000 or 1000 cells per injection. Tumor growth was monitored for 1 month. After 1 month, mice were euthanized and tumors were harvested for the subsequent volume measurement. Tumor volume was calculated with the formula: ¼ [(length) (width) (width)]/2. For the tail vein injection experiment, 1 × 10^5^ inhibitor‐treated or DMSO‐treated CU110 cells were intravenously injected into lateral tail veins of C57BL/6 mice. After 3 weeks, the mice were euthanized and their lung tissues were collected for gross assessment and hematoxylin/eosin (H&E) staining.

### 2.12 Statistical analysis

Statistical calculations were performed using prism 5 software (GraphPad Software Inc., San Diego, CA, USA). Data were presented as the mean ± SD as indicated in the figure legends. The statistical significance of quantitative data was determined using the two‐tailed Student *t‐*test, and results were presented as *P*‐values (*P < 0.05, **P < 0.01).

## 3 Results

### 3.1 Overexpression of PIK3CA promotes EMT and enriches head and neck CSCs

**Figure 1:**
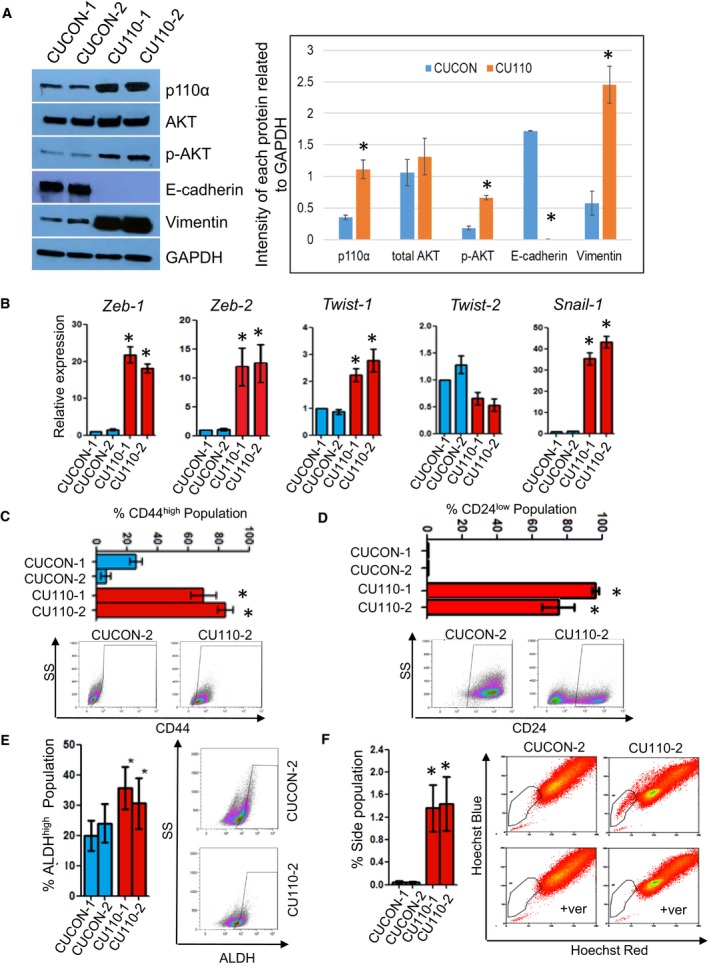
Overexpression of *PIK3CA* promotes EMT and enriches CSCs. (A) Western blotting of p110α, total AKT, phospho‐AKT473, E‐cadherin and vimentin in two CUCON and two CU110 cells. GAPDH was used as a loading control. Quantification of western blots by imagej is shown on the right. *P < 0.05 (two‐tailed Student *t*‐test). (B) qRT‐PCR analysis of transcriptional factors regulating EMT in two CUCON and two CU110 cells. The results are presented as mean of two different experiments with SD (error bars). Each experiment was done in triplicate. *P < 0.05 (two‐tailed Student *t*‐test). (C) FACS analysis of cell surface marker, CD44 in two CUCON and two CU110 cells. Quantification data are represented as mean ± SD (*n* = 3). An example of CD44 FACS is shown on the right. *P < 0.05 (two‐tailed Student *t*‐test). (D) FACS analysis of cell surface marker, CD24 in two CUCON and two CU110 cells. Quantification data are represented as mean ± SD (*n* = 3). An example of CD44 FACS is shown on the right. *P < 0.05 (two‐tailed Student *t*‐test). (E) ALDH activity assay using ALDEFLUOR staining in two CUCON cells and two CU110 cells; quantification data are represented as mean ± SD (*n* = 3). *P ˂ 0.05. An example of ALDH FACS is shown on the right. *P < 0.05 (two‐tailed Student *t‐*test). (F) SP fraction detected by Hoechst dye‐effluxing assay in two CUCON and two CU110 cells. Right panel: representative FACS plots of CUCON and CU110 cells treated with Hoechst 33342 in the presence and absence of verapamil (ver). The specificity of SP fraction was validated by a verapamil elimination experiment. Left panel: quantification of gated SPs of CUCON and CU110 cells. Quantification data are represented as mean ± SD (*n* = 3). *P < 0.05 (two‐tailed Student *t‐*test).

We have recently reported a GEMM in which the *PIK3CA* transgene is overexpressed in murine head and neck epithelia (Du *et al.*, 2016). Although overexpression of *PIK3CA* alone is not sufficient for HNSCC development; it significantly promotes HNSCC invasion and metastasis. EMT and enriched CSC phenotypes were observed in the 4NQO‐induced tumor from the *PIK3CA* mice (hereafter referred to as the *PIK3CA‐*tumors) in comparison with the 4‐NQO‐induced tumors from the control mice (hereafter referred to as the control‐tumors) (Du *et al.*, 2016). We further established primary murine HNSCC cell lines from two individual *PIK3CA*‐tumors (hereafter referred to as CU110‐1 and CU110‐2) and two individual control‐tumors (hereafter referred to as CUCON‐1 and CUCON‐2) (Du *et al.*, 2016) to facilitate studying of the molecular mechanisms underlying the *PIK3CA*‐driven EMT and CSC characteristics. As anticipated, the resulting CU110 cells showed higher expression of p110α and enhanced phosphorylation of AKT level compared with CUCON cells (Fig. 1A), indicating that PI3K/AKT signaling is highly active in the cell lines derived from tumors of *PIK3CA*‐GEMM.

We then characterized these cell lines to determine whether they recapitulate EMT phenotype and enrichment of CSC population as seen in the primary tumors they were derived from. Western blotting results showed that overexpression of *PIK3CA* in CU110 cells promoted EMT evidenced by loss of the epithelial marker, E‐cadherin, and overexpression of the mesenchymal marker, vimentin (Fig. 1A). IF staining for E‐cadherin and vimentin further confirmed the EMT phenotype in CU110 cells compared with CUCON cells (Fig. S1A). Next, we analyzed expression of various transcription factors known to regulate the EMT program using quantitative RT‐PCR (qRT‐PCR). We found that Zeb1, Zeb2, Twist1 and Snail expression was significantly higher in CU110 cells than CUCON cells (Fig. 1B). As the EMT process generates poorly differentiated tumor cells defined by cytokeratin loss (Brabletz, 2012b), we further assessed expression of a panel of cytokeratins in both CU110 and CUCON cells. We found that multiple cytokeratins, except K8, were downregulated in CU110 cells compared with the CUCON cells, reflecting a general de‐differentiation and EMT state (Fig. S1B). K8 is a marker for spindle cell carcinoma, a pathological example of EMT (Lu *et al.*, 2006; Miettinen, 1991; Zidar *et al.*, 2018), and is often increased in late stage SCC (Makino *et al.*, 2009). These results further support an EMT and poorly differentiated phenotype of CU110 cells. Together, these data demonstrate that overexpression of *PIK3CA* promotes EMT, and de‐differentiation.

It has been shown that EMT and de‐differentiation generate cells possessing properties of stem cells and confer cancer cells with stem cell‐like characteristics (Mani *et al.*, 2008). To examine whether the EMT and de‐differentiation phenotypes of CU110 cells enrich CSCs, we utilized several putative surface markers reported for CSC isolation in HNSCC, including CD44^high^, CD24^low^, ALDH^high^ and SP (Chen *et al.*, 2011a; Clay *et al.*, 2010; Prince *et al.*, 2007; Tabor *et al.*, 2011; Todoroki *et al.*, 2016). FACS results showed significantly higher numbers of CD44^high^ (Fig. 1C) and CD24^low^ cells (Fig. 1D), increased ALDH activity (Fig. 1E) and SP fraction (Fig. 1F) in CU110 cells compared with CUCON cells.

We observed enrichment of CSC population in CU110 cells derived from the *PIK3CA*‐tumors. One question is whether the tumor‐derived cell lines represent the true situation in the tumor tissues from which they originally derived, since multicellular communication in tumor tissues may affect properties of CSCs. We have shown increased expression of multiple CSC markers, including CD44, CD166, CD24, Nanog, Oct4 and EZH2, in the *PIK3CA‐*tumors than the control‐tumors in our previous report (Du *et al.*, 2016). Here, we further examined CD44, SOX2 (Lee *et al.*, 2014) and BMI1 (Chen *et al.*, 2011b) by IHC in the *PIK3CA‐*tumors and the control‐tumors (Fig. S1C). CD44‐positive cells (membrane staining) were distributed in nearly the entire field of the *PIK3CA* tumors compared with those in the control‐tumors, where CD44‐positive cells were only seen around the edge of the tumors (Fig. S1C, left). SOX2‐positive cells (nuclear staining) were also significantly increased in the *PIK3CA‐*tumors compared with in the control‐tumors (45.50 ± 5.97 positive cells per field, vs 8.25 ± 1.89 positive cells per field; *P* < 0.05, Fig. S1C, Middle). Nuclear staining of BMI1 was seen in both the *PIK3CA and* the control‐tumors. However, staining was much stronger in the *PIK3CA*‐tumors than in the control*‐*tumors (Fig. S1C, right). In combination with our previous report and present results, we showed that overexpression of PIK3CA enriches CSCs both in the primary tumor tissues of *PIK3CA* mice and in the cell lines derived from these tumors.

### 3.2 Sphere‐forming capacity is a functional measurement for cancer stemness properties of HNSCC

**Figure 2:**
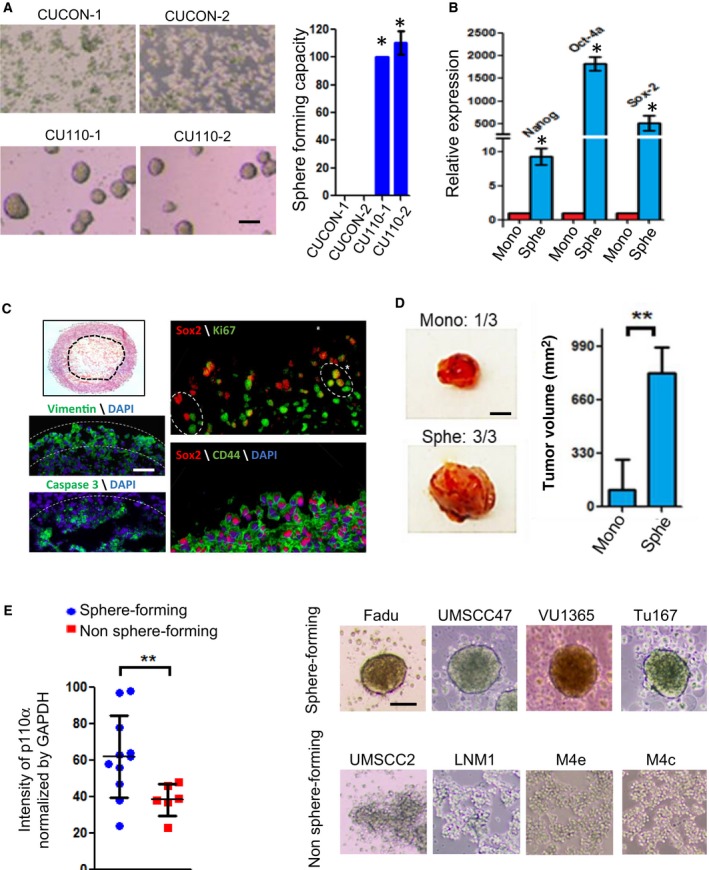
Sphe‐forming is a functional readout of cancer stemness. (A) HNSCC Sphe‐forming ability of two CUCON and two CU110 cells. Left panel: Phase‐contrast images of CUCON and CU110 cells in serum‐free and ultralow attachment culture condition. Right panel: Quantification of Sphe numbers for two CUCON and two CU110 cells. Sphes with diameter ≥ 30 μm were counted. Sphe‐forming capacity is defined as percentage of total number of Sphe formed by CU110 cells in comparison with CUCON cells. Quantification data are represented as mean ± SD (*n* = 3). *P < 0.05 (two‐tailed Student *t‐*test). Scale bar: 100 µm. (B) qRT‐PCR for expression of embryonic stem cell genes in the mono‐ or Sphe‐cultured CU110 cells. GAPDH was used as an internal control. The results are presented as mean of two different experiments with SD (error bars). Each experiment was done in triplicate. *P < 0.05 (two‐tailed Student *t‐*test). (C) Left panel (top): H&E staining for HNSCC Sphe section; left panel (bottom): vimentin‐positive layer was delineated by white dotted lines and hollow center of spheroid was delineated by white dotted lines. Right panel: IF staining on Sphe sections using the antibodies as indicated in figures. Scale bar: 50 µm. (D) *In vivo* tumorigenicity by subcutaneous injection of 1000 cells isolated from either Sphe‐ or mono‐cultured CU110 cells to the flanks of C57BL6 mice. Left panel: Images of tumors developed from mono (one of three mice) or Sphe‐cultured CU110 cells (three of three mice). The tumor volume is average of tumors developed in mice with SD for each group and is shown on the right. **P ˂ 0.01 (two‐tailed Student *t‐*test). Scale bar: 2 mm. (E) Correlation between p110α expression and Sphe‐forming ability in human HNSCC cell lines. Left panel: Western blotting and HNSCC Sphe‐forming assay were performed in 17 human head and neck cancer cell lines (UMSCC1/2/10A/10B/22A/22B/47, VU1131/1365, SCC9, Fadu, HN6, Tu167, Cal27, M4C/4E and LNM1). Relative quantification of p110α was done by quantifying band intensity of p110α and GAPDH, and is shown as percentage of GAPDH band intensity. **P ˂ 0.01 (two‐tailed Student *t‐*test). Right panel: Representative images of Sphe‐forming capacity of HNSCC cell lines in serum‐free and ultralow attachment culture condition. Scale bar: 100 µm.

To determine whether the enriched CSC population of CU110 cells exhibit efficient self‐renewal, we performed a Sphe‐forming assay using CU110 and CUCON cells. Consistent with the enrichment of CSCs revealed by multiple surface markers in Fig. 1C–F, the CU110 cells, but not CUCON cells, were able to form tumor Sphes (Fig. 2A). To further examine whether the Sphe possess CSC properties, we measured mRNA expression of embryonic stem cell markers Nanog, Oct4 and Sox2. As shown in Fig. 2B, these markers were significantly overexpressed in Sphe compared with mono‐cultured CU110 cells. We then examined two additional CSC markers, ALDH1A1 and ABCG2; both molecules were significantly increased in Sphe compared with mono (Fig. S2). We further performed H&E staining on sections of spheroids and observed distinguishable layers with a hollow center. Moreover, the inner layers of these spheroids featured intensive extracellular matrix (Fig. 2C, left top panel). Consistent with higher mRNA expression of Sox2 in spheroids, the outer layer contained many SOX2^+^ cells observed by IF (Fig. 2C, right top panel). However, not all CD44^+^ cells were SOX2^+^ (Fig. 2C, right lower panel). Moreover, some SOX2^+^ cells were Ki67^+^ (Fig. 2C, right top panel), suggesting that proliferation of CSCs may be heterogeneous and/or dynamic (Brown *et al.*, 2017). In addition, almost all outer‐layer cells were Vimentin‐positive and cells near the hollow center showed caspase 3‐positive staining, indicating that these cells were undergoing apoptosis (Fig. 2C, lower left panel).

To evaluate tumorigenicity of spheral cells *in vivo*, we injected 1000 cells from either Sphe‐ or mono‐cultured CU110 cells into the flanks of syngeneic C57BL6 mice. All Sphe‐cultured CU110 cells generated tumors, but only one mouse developed a smaller tumor for mono‐cultured CU110 cells. The average volume of tumors from Sphes was significantly greater than that from the tumor of mono cells (Fig. 2D). Last, we investigated whether Sphe‐forming ability is correlated with p110α expression in human HNSCC cell lines. Among the 17 human HNSCC cell lines we screened (Table S3), the Sphe‐forming ability was found to be well correlated with p110α expression levels, suggesting that overexpression of PIK3CA enriches CSC population in human HNSCC as well (Fig. 2E, Table S3).

### 3.3 Knocking down of *PIK3CA* failed to reverse EMT or reduce the CSC pool

**Figure 3:**
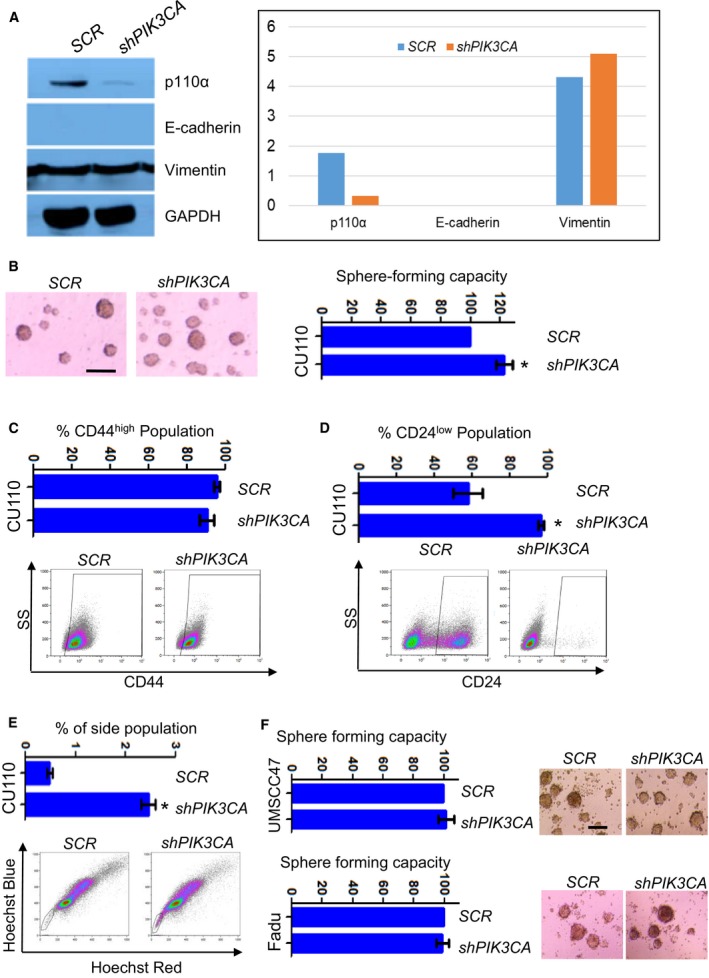
Knocking down of *PIK3CA* failed to reverse EMT and reduce CSC population. (A) Western blotting of p110α, E‐cadherin and vimentin in CU110‐2 cells stably transfected with lentiviral‐mediated shRNA (*shPIK3CA*) or a scrambled control (*SCR*). GAPDH was used as a loading control. Quantitation of western blots is shown in right. (B) HNSCC Sphe‐forming assay of CU110 cells stably transfected either *shPIK3CA* or *SCR* lentivirus. Sphes with diameter ≥ 30 μm were counted; quantification of Sphe is shown on the right, *n* = 3; error bars indicate SD. *P < 0.05 (two‐tailed Student *t‐*test). Scale bar: 100 µm. (C) FACS analysis of CD44 in CU110 cells stably transfected either *shPIK3CA* or *SCR* lentivirus. Quantification of CD44 population is shown on top. *n* = 3; error bars indicate SD. (D) FACS analysis of CD24 in CU110 cells stably transfected either *shPIK3CA* or *SCR* lentivirus. Quantification of CD24 population is shown on top. *n* = 3; error bars indicate SD. (E) SP fraction using Hoechst dye‐effluxing analysis in CU110 cells stably transfected either *shPIK3CA* or *SCR* lentivirus. Quantification of SP fraction is shown on top. *n* = 3; error bars indicate SD. *P < 0.05 (two‐tailed Student *t‐*test). (F) HNSCC Sphe‐forming assay of Fadu or UMSCC47 cell lines stably transfected with either *shPIK3CA* or *SCR*. The quantification is shown on the left. Error bars indicate SD. Scale bar: 100 µm.

Next, we examined whether *PIK3CA* is required to maintain an EMT phenotype or CSC populations in the *PIK3CA*‐overexpressing CU110 cells. We stably knocked down *PIK3CA* expression in CU110 cells using shRNA against *PIK3CA*. Surprisingly, the knocking down of *PIK3CA* failed to induce E‐cadherin or reduce vimentin expression, as shown by western blot and IF staining (Figs 3A and S3), indicating failure to reverse EMT in the *PIK3CA*‐overexpressing cells. We further examined the effects of knocking down *PIK3CA* on CSCs in CU110 cells. As shown in Fig. 3B–E, instead of reducing CSC numbers, knocking down of *PIK3CA* increased the CSC pool in CU110 cells. In the Sphe‐forming assay, knocking down *PIK3CA* moderately increased Sphe numbers (Fig. 3B). In the CSC surface marker assays, although knocking down of *PIK3CA* had no effects on the CD44^high^ population (Fig. 3C), it significantly increased CD24^low^ populations (Fig. 3D). Furthermore, knocking down of *PIK3CA* caused about a fivefold increase in SP fraction (Fig. 3E).

To examine whether our findings in the mouse model applied to human HNSCCs, we screened 17 human HNSCC cell lines and chose UMSCC47, which expressed the highest level of *PIK3CA* among these lines (Fig. S4A). UMSCC47 was derived from a HPV^+^ tongue SCC (Brenner *et al.*, 2010). To compare overexpression and gain‐of‐function mutations which also occur in human HNSCCs, we chose the Fadu cell line, which harbors an H1047R hot‐spot mutation in *PIK3CA* and was derived from an HPV^–^ hypopharynx SCC (Qiu *et al.*, 2006). Knocking down of *PIK3CA* in these two human HNSCC cell lines reduced cell proliferation (Fig. S4B,C). However, similar to the effect we observed in the murine CU110 cells, knocking down of *PIK3CA* failed to reduce Sphe‐forming ability in either UMSCC47 or Fadu cell lines (Fig. 3F). Thus, it appears that although PI3K amplification promotes EMT and CSCs, blocking it, cannot reverse this phenotype.

### 3.4 Knockdown of key components in PI3K pathway promotes CSC population

**Figure 4:**
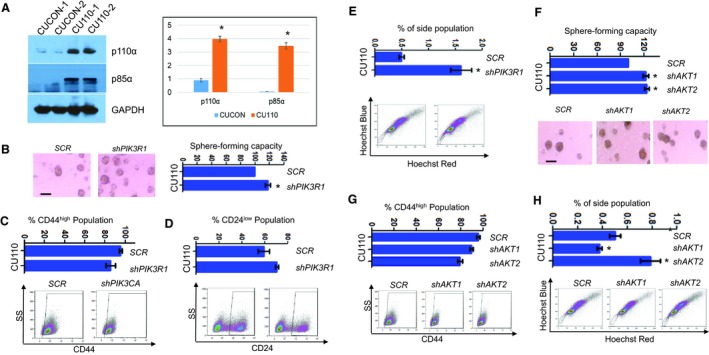
Knocking down of key components in PI3K pathway promotes CSC population. (A) Western blotting of p110α and p85α in two CUCON and two CU110 cells. GAPDH was used as a loading control. Quantitation of western blots is shown on the right. *P < 0.05 (two‐tailed Student *t‐*test). (B) HNSCC Sphe‐forming assay of CU110 cells stably transfected with either *shPIK3R1* or *SCR* lentivirus. The quantification is shown on top. Sphes with diameter ≥ 30 μm were counted. *n* = 3; error bars indicate SD. *P < 0.05 (two‐tailed Student *t‐*test). Scale bar: 100 µm. (C) FACS analysis of CD44 population in CU110 cells stably transfected with either *shPIK3R1* or *SCR* lentivirus. Error bars indicate SD. *n* = 3. (D) FACS analysis of CD24 population in CU110 cells stably transfected with either *shPIK3R1* or *SCR* lentivirus. Error bars indicate SD. *n* = 3. (E) SP fraction using Hoechst dye‐effluxing analysis in CU110 cells stably transfected either *shPIK3R1* or *SCR* lentivirus. Quantification of SP fraction is shown on top. Error bars indicate SD. *n* = 3. *P < 0.05 (two‐tailed Student *t‐*test). (F) HNSCC Sphe‐forming assay of CU110 cells stably transfected with *shAKT1*, *shAKT2* or *SCR* lentivirus. The quantification is shown on top. Sphes with diameter ≥ 30 μm were counted. *n* = 3; error bars indicate SD. *P < 0.05 (two‐tailed Student *t‐*test). Scale bar: 100 µm. (G) FACS analysis of CD44 population in CU110 cells stably transfected with *shAKT1*, *shAKT2* or *SCR* lentivirus. Error bars indicate SD. *n* = 3. (H) SP fraction using Hoechst dye‐effluxing analysis in CU110 cells stably transfected *shAKT1*, *shAKT2* or *SCR* lentivirus. Quantification of SP fraction is shown on top. Error bars indicate SD. *n* = 3. *P < 0.05 (two‐tailed Student *t‐*test).

The failure to reduce CSC pool upon *PIK3CA* knockdown was unexpected and prompted us to examine the effect of knockdown on other key components in PI3K pathway. We first examined the expression levels of several PI3K isoforms in *PIK3CA*‐overexpressing cells. As shown in Figs S5A and 4A, the gene encoding the regulatory subunit of PI3K (*PIK3R1*), the gene encoding for the p85α subunit of PI3K, is concurrently highly expressed in CU110 cells, as measured by qRT‐PCR and western blotting. Interestingly, the p85α expression also correlated with Sphe‐forming capacity in human HNSCC cell lines (Fig. S5B). To further evaluate the relevance of this, we stably knocked down *PIK3R1* in CU110 cells (Fig. S5C). Similar to the effect of knockdown of *PIK3CA*, genetic suppression of *PIK3R1* was not able to reverse EMT, as shown by qRT‐PCR of E‐cadherin, vimentin and other transcriptional factors regulating EMT (Fig. S5D). Knockdown of *PIK3R1* also failed to reduce the CSC pool by Sphe‐forming assays (Fig. 4B), FACS analysis of CD44 and CD24 (Fig 4C,D), and SP fraction (Fig. 4E). Instead, similar to *PIK3CA* knockdown, knockdown of *PIK3R1* increased the Sphe formation (Fig. 4B).

It has been reported that the ratio of *AKT1* and *AKT2* expression regulates EMT and CSC properties in a breast cancer model (Iliopoulos *et al.*, 2009). Thus, we further knocked down *AKT1* or *AKT2* in the CU110 cells (Fig. S5E). Similar to the effects of *PIK3CA* or *PIK3R1* knockdown, genetic suppression of *AKT1* or *AKT2* had no effects on EMT, as shown by qRT‐PCR of *E‐cadherin*, *vimentin* and several transcriptional factors regulating EMT (Fig. S5F). Knockdown of either *AKT1* or *AKT2* also failed to decrease the CSC population, as shown by Sphe‐forming assay (Fig. 4F), FACS analysis of surface markers CD44 (Fig. 4G) and SP fraction (Fig. 4H).

### 3.5 Targeting multiple receptor tyrosine kinase pathways effectively ablates CSC population by inhibiting Ephs, TRKs and c‐Kit

**Figure 5:**
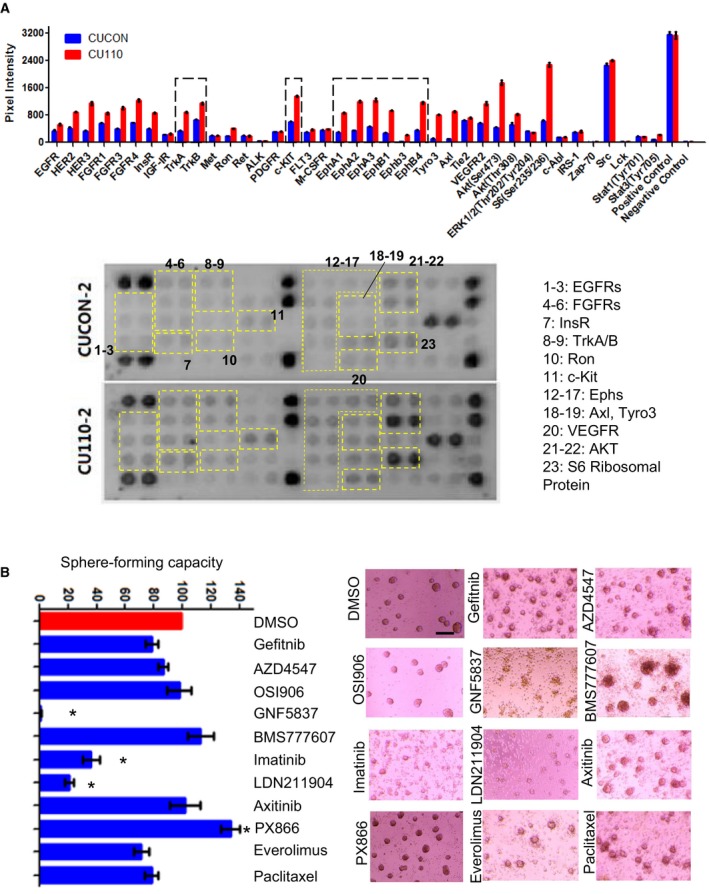
Identifying multiple RTK pathways effectively eliminates CSC populations. (A) RTK protein array in the *PIK3CA*‐overexpressing cells (CU110) and control cells (CUCON) using the PathScan RTK Signaling Antibody Array Assay Kit. Top panel: quantification, lower panel: chemiluminescent images. (B) Screening for pharmaceutical inhibitors effectively reducing CSC population in CU110 cells using HNSCC Sphe‐forming assay. The quantification is shown on the left. Error bars indicate SD. *n* = 3. *P < 0.05 (two‐tailed Student *t‐*test). Scale bar: 100 µm.

Since *PIK3CA*‐induced CSC properties are no longer dependent on PI3K signaling, we sought to identify other mechanisms important for maintaining CSC population. Emerging evidence suggests a critical role of RTKs in maintaining CSC phenotypes (Cheng *et al.*, 2016). We thus performed a RTK protein array to compare the differences between the *PIK3CA*‐overexpressing CU110 cells and the CUCON control cells. As shown in Fig. 5A, compared with CUCON cells, there were multiple activated RTKs in CU110 cells: EGFR, FGFRs, InsR, TRKA and B, c‐Kit, Ephs, Tyro3, Axl, VEGFRs, S6 ribosomal protein, AKT.

**Figure 6:**
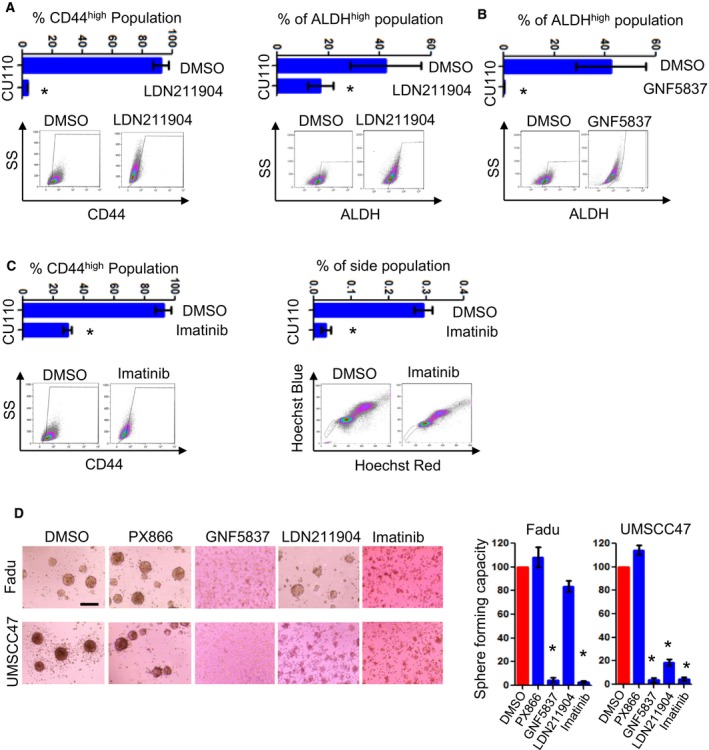
Targeting multiple RTK pathways effectively eliminates CSC populations with inhibiting Ephs, TRKs, and c‐Kit the most prominent. (A) FACS analysis of CD44 (left) or ALDH (right) in CU110 cells treated with LDN211904. Error bars indicate SD. *n* = 3. *P < 0.05 (two‐tailed Student *t‐*test). (B) FACS analysis of ALDH in CU110 cells treated with GNF5837. Error bars indicate SD. *n* = 3. *P < 0.05 (two‐tailed Student *t‐*test). (C) FACS analysis of CD44 (left) or SP fraction (right) in CU110 cells treated with imatinib. Error bars indicate SD. *n* = 3. *P < 0.05 (two‐tailed Student *t‐*test). (D) Effect of pharmaceutical inhibitors (as indicated in the figure) on reducing CSC population in human HNSCC cell lines: Fadu and UMSCC47, using Sphe‐forming assay. The quantification is shown on right. *n* = 3; error bars indicate SD. *P < 0.05 (two‐tailed Student *t‐*test). Scale bar: 100 µm.

To further identify key RTKs maintaining CSC phenotype, we utilized a pharmaceutical inhibitor screen approach to assess HNSCC Sphe formation. We selected inhibitors targeting the activated RTK pathways from the RTK protein array experiment described above. In addition, chemotherapy drug paclitaxel was also included to assess its effects on Sphe‐forming ability. We have determined IC_50_ for each agent in CU110 cells (Table S4) and used this dose to determine the effect of each agent on Sphe‐forming ability of CU110 cells. As shown in Fig. 5B, PX866 increased the numbers of HNSCC Sphes, which is consistent with the result of genetic inhibition of PI3K pathway in Figs 3 and 4, suggesting that inhibition of PI3K pathway may elicit a feedback mechanism of increasing RTKs to maintain CSC property in compensation. In contrast, inhibitors LDN211904 (targeting Ephs), GNF5837 (targeting TRKA/B) and Imatinib (targeting c‐kit) effectively reduced or ablated Sphe formation. In addition, treating CU110 with LDN211904 also reduced CD44^high^ and ALDH^high^ populations (Fig. 6A), treating CU110 cell with GNF5837 significantly reduced ALDH^high^ populations (Fig. 6B), and treating CU110 with imatinib reduced CD44^high^ and SP fraction (Fig. 6C). To further validate this result in human HNSCC cell lines, we treated UMSCC47 and Fadu cells with PX866, LDN211904, GNF5837 and imatinib. Consistent with the results from CU110 cells, compared with PX866, treatment with LDN211904, GNF5837 and imatinib inhibited Sphe formation in UMSCC47 and Fadu cell lines (Fig. 6D).

### 3.6 Ponatinib, a multi‐RTK inhibitor that targets Ephs, TRKs and c‐Kit superiorly eliminates CSCs induced by overexpression of PIK3CA

**Figure 7:**
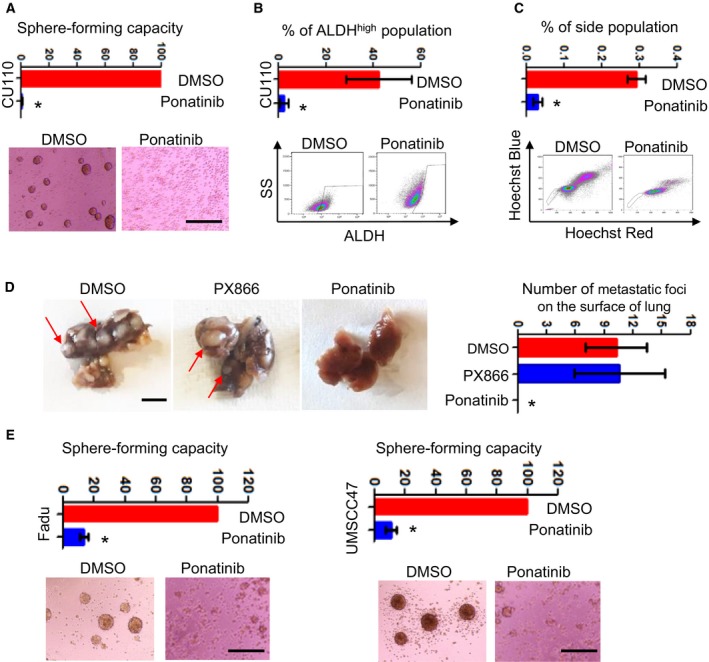
Ponatinib, a multi‐kinase inhibitor targeting Ephs, TRKs and c‐Kit, effectively eliminates CSC population in HNSCC. (A) HNSCC Sphe‐forming assay of CU110 cells treated with ponatinib. The quantification is shown on top. Sphes with diameter ≥ 30 μm were counted. *n* = 3; error bars indicate SD. *P < 0.05 (two‐tailed Student *t‐*test). Scale bar: 100 µm. (B) FACS analysis of ALDH in CU110 cells treated with ponatinib. The quantification is shown on top. *n* = 3; error bars indicate SD. *P < 0.05 (two‐tailed Student *t‐*test). (C) FACS analysis of SP fraction in CU110 treated with ponatinib (left). The quantification is shown on top. *n* = 3; error bars indicate SD. *P < 0.05 (two‐tailed Student *t‐*test). (D) Left: Gross pictures of lung tissues harvested from mice received tail vein injection of CU110 cells treated with DMSO, PX866 or ponatinib. Red arrow indicates metastatic foci on the surface of the lung. Right: Relevant quantification of the metastatic foci on the surface of lung. *n* = 4; error bars indicate SD. *P < 0.05 (two‐tailed Student *t‐*test). Scale bar: 100 µm. (E) Effect of ponatinib on reducing CSC population in human HNSCC cell lines: Fadu and UMSCC47, using Sphe‐forming assay. The quantification is shown on the right. *n* = 3; error bars indicate SD. *P < 0.05 (two‐tailed Student *t‐*test). Scale bar: 100 µm.

The attenuation of CSC phenotype upon inhibition of Ephs, TRKs and c‐Kit separately led us to hypothesize that inhibitor(s) co‐targeting all three molecules may have additive effects on the CSC phenotype. Ponatinib was chosen because it inhibits multiple RTKs, particularly Ephs and TRKs below the 50‐nm level (O'Hare *et al.*, 2009). We treated CU110 cells in a Sphe‐forming assay with ponatinib to test this and found nearly complete elimination of Sphe formation, ALDH^high^ population and SP fraction (Fig. 7A‐C). To assess the *in vivo* effect of ponatinib treatment on HNSCC metastasis, we treated CU110 cells with DMSO, PX866 or ponatinib for 48 h. Then, 1 × 10^5^ viable cells after treatment were injected into the tail vein of syngeneic C57BL6 mice. After 21 days, lung tissues were harvested from these three groups of mice. As shown in Fig. 7D, CU110 cells or cells treated with PX866 generated multiple lung nodules. In contrast, none of the lungs had visible lung modules in the CU110 cells treated with ponatinib. To further validate this result in human HNSCC cell lines, we treated UMSCC47 and Fadu cells with ponatinib. Consistent with the results from CU110 cells, ponatinib treatment significantly reduced the number of Sphes formed (Fig. 7E).

## 4 Discussion

Emerging evidence suggests that PIK3CA has a vital role in regulating EMT and CSC properties in a variety of cancers. For example, *PIK3CA* mutation is most frequently seen (nearly 50%) in aggressive breast cancers that display EMT and CSC properties (Hennessy *et al.*, 2009). Recent studies using breast cancer mouse models demonstrate that expression of *PIK3CA^H1047R^* in mammary glands developed multipotent lineage of breast cancer, conferring intra‐ or inter‐tumor heterogeneity (Koren *et al.*, 2015; Van Keymeulen *et al.*, 2015). Interestingly, oncogenic PIK3CA engineered in induced pluripotent stem cells caused partial loss of epithelial morphology and up‐regulation of stemness markers (Madsen *et al.*, 2019). In line with our previous report, this study demonstrates a key role for *PIK3CA* in driving EMT and CSC phenotypes during cancer progression.

The interplay of EMT, CSCs and metastasis is inevitably tissue‐ and context‐specific. Although EMT is the process by which cell acquire plasticity and gain the properties of stem cells (Brabletz, 2012a; Gupta *et al.*, 2019; Mani *et al.*, 2008), recent studies suggest that metastasis can be independent of EMT, and metastatic CSCs do not necessarily have a mesenchymal‐like origin (Fischer *et al.*, 2015; Pascual *et al.*, 2017; Zheng *et al.*, 2015). Accordingly, our data show that eliminating CSC and blocking metastasis does not necessarily accompany complete reversal of the EMT phenotype, indicating that CSC and metastatic traits can be uncoupled from EMT. Indeed, a non‐mesenchymal, epithelial‐like population has recently been shown to confer CSC and metastatic traits that drive HNSCC progression (Pascual *et al.*, 2017). Considering the complexity among EMT, CSCs and metastatic traits, it is still conceptually and fundamentally important to identify and interrogate such a relationship and its driven outcome in local invasiveness and distant metastasis.

It was unexpected that inhibition of PI3K pathway failed to reverse either EMT or CSC phenotypes. PI3K pathway has been shown as a promising target in pre‐clinical studies, but the therapeutic efficacy is modest as a single agent in clinical trials (Hanker *et al.*, 2019; Janku *et al.*, 2018). In light of our observations, one of the reasons might be insufficient inhibition of EMT and CSC when these traits become independent of PI3K signaling. The ratio of *AKT1* and *AKT2* has been reported to regulate EMT and CSC traits through regulation of miR‐200s in breast cancer (Iliopoulos *et al.*, 2009). In addition, we found that the level of PI3K family member *PIK3R1* is increased in the *PIK3CA*‐overexpressing HNSCCs. However, knockdown of *AKT1*, *AKT2* or *PIK3R1* did not reverse EMT or reduce the CSC population. Instead, we found that it genetically suppressing major components of the PI3K pathway or, paradoxically, pharmacologically targeting PI3K appeared to promote CSC population in the *PIK3CA*‐overexpressing HNSCCs. Although it is not clear why interfering PI3K signaling would promote CSC traits, we suspect that activation of alternative pathway or feedback loop(s) could be one of the explanations. For example, a fraction of PI3K mutant mammary tumors escape PI3K dependence by compensatory activation of MEK‐ERK signaling (Cheng *et al.*, 2016). Another example is that inhibition of mTOR in PI3K mutant cells results in activation of FGFR1‐dependent Notch signaling to confer CSC survival (Bhola *et al.*, 2016). Inhibition of EGFR or chemotherapy using cisplatin was also reported to enhance CSC population (Arasada *et al.*, 2014; Nor *et al.*, 2014; Shien *et al.*, 2013). In these cases, inhibiting one major target/pathway resulted in the activation of another target/pathway, providing an advantage for CSC survival.

To identify the compensatory mechanisms of PI3K‐independency, we screened RTKs, as they interact highly with the PI3K pathway. We found that multiple RTKs were activated in the *PIK3CA*‐overexpressing HNSCCs, with Ephs, TRKs and c‐Kit signaling the most prominent. Ephs are highly expressed in normal stem cells and in CSCs in breast, ovary, lung, glioma and melanoma, and are associated with tumor growth and metastasis (Murai and Pasquale, 2010; Pasquale, 2010). Besides, EphA4 receptor is overexpressed in the EMT/stem‐like breast cancer cells (Lu *et al.*, 2014) and Ephrin B2 is associated with progression and resistance to chemotherapy and radiation therapy in HNSCC (Oweida *et al.*, 2017). Mutations and gene fusions in TRKs have also been reported in multiple cancers, including ovarian, colorectal, melanoma and lung (Vaishnavi *et al.*, 2015). TRKB has been reported to suppress anoikis and induce metastasis (Douma *et al.*, 2004). In HNSCC, TRKB has been shown to induce EMT and promote cancer progression (Kupferman *et al.*, 2010). In addition, TRK has also been shown to promote brain CSCs in malignant glioma (Lawn *et al.*, 2015). c‐Kit has been used as a stem cell marker for hematopoietic stem cells and has also been shown to be unregulated in CSCs. For example, targeting c‐Kit reduced CSC numbers in lung cancer (Levina *et al.*, 2010). In line with these studies, we found that Ephs, TRKs or c‐Kit contributes to maintenance of *PIK3CA* overexpression‐induced CSC traits. However, how overexpression of PIK3CA activates these signaling pathways, compensating for the maintenance of CSC trait, remains under investigation. Our study showed that ponatinib, the inhibitor targeting multiple RTKs, including Eph, TRK and c‐Kit, was the most potent inhibition of CSC population than inhibition of Eph, TRK or c‐Kit individually. This result suggests that co‐targeting of these signaling pathways can achieve optimal effects. It also suggests that ponatinib could be one of the candidates for treatment of HNSCC recurrence and metastasis, as the drug has been approved and used for leukemia patients, who tolerate it well (Poch Martell *et al.*, 2016).

## 5 Conclusion

In conclusion, our results show that *PIK3CA* overexpression enriches CSC population in both murine and human HNSCCs. However, the maintenance of CSC population in *PIK3CA*‐overexpressing HNSCC becomes PI3K pathway‐independent. Multiple RTK activation, particularly Ephs, TRKs and c‐Kit signaling, may serve as compensatory mechanisms to maintain CSC population in *PIK3CA*‐overexpressing HNSCC. Thus, co‐targeting Ephs, TRKs and the c‐Kit pathway may be effective in eliminating the PI3K‐independent CSC population, providing potential targets for future development of a novel anti‐CSC therapeutic approach for HNSCC patients, particularly in patients with *PIK3CA* amplification.

## Conflict of interest

The authors declare no conflict of interest.

## Author contributions

XC and SLL designed the study. XC, YC, WS, LL, YL, HW and MO carried out and interpreted experiments. SB, SS and JS were involved in data analysis and interpretation. XC, WS, SB and SLL wrote the manuscript with contributions and approval from all authors.

## Supporting information


**Fig. S1.** Overexpression of PIK3CA promotes EMT and de‐differentiation. (A) Immunofluorescence staining of E‐cadherin (green) and vimentin (red) in two CUCON and two CU110 cell lines. DAPI stain (blue) was used to visualize the nucleus. Scale bar: 50 µm. (B) qRT‐PCR analysis of cytokeratins in CUCON and CU110 cell lines. The results are presented as mean of two different experiments with standard deviations (error bar). Each experiment was done in triplicate. *P < 0.05. (C) Immunohistochemistry staining of CD44 (left), SOX2 (middle), and BMI1 (right) in the 4NQO‐induced tumors from either the *PIK3CA* or control mice; *n* = 3 for each group. SOX2^+^ cells were enumerated in three independent fields from three *PIK3CA‐*tumors and three control‐tumors. The quantitation result is included in the text. Scale bar: 50 µm (CD44 and BMI1), 25 µm (SOX2).
**Fig. S2.** qRT‐PCR analysis of ALDH1A1 and ABCG2 in CU110 cells cultured in either monolayer or sphere. The results are presented as mean of two different experiments with standard deviations (error bar); Each experiment was done in triplicate. *P < 0.05.
**Fig. S3.** Knocking down of PIK3CA failed to reverse EMT. Immunofluorescence staining of E‐cadherin or vimentin in CU110‐2 cells stably transfected with either shPIK3CA or a scrambled control (SCR). DAPI staining (blue) was used to visualize the nucleus. Scale bar: 50 µm.
**Fig. S4.** Effect of knocking down of *PIK3CA* on human head and neck cancer cell lines. (A) *PIK3CA* expression in 17 human head and neck cancer cell lines (as indicated) using qRT‐PCR. Experiment was done in triplicate. (B) Western blotting of p110α, AKT, pAKT(Ser473) and GAPDH in Fadu (left) and UMSCC47 cell lines (right) stably transfected with either lentiviral‐mediated *shPIK3CA* or *SCR*. Quantitation of western blots is shown on the right. (C) Cell proliferation assay of Fadu (left) and UMSCC47 (right) cell lines stably transfected with either* shPIK3CA* or *SCR*. *n *= 3; error bars indicate SD. *P < 0.05.
**Fig. S5.** Examination of PI3K isoforms in murine HNSCC cells and evaluation of knockdown efficiency. (A) qRT‐PCR results of PI3K isoforms in two CUCON and two CU110 cells. Fold changes are relative to CUCON‐1 cells; GAPDH was used as an internal control. *n *= 3; error bars indicate SD. The results are presented as mean of two different experiments with SD (error bar). Each experiment was done in triplicate. *P < 0.05. (B) Correlation between p85α protein and sphere‐forming ability in human HNSCC cell lines. Western blotting and HNSCC sphere‐forming assay were performed in 12 human head and neck cancer cell lines (VU1131, VU1365, UMSCC1, UMSCC2, UMSCC47, Fadu, Tu167, HN6, LNM1, CAL27, M4C, and M4E). Relative quantification of p85α was done by quantifying band intensity of p85α and GAPDH, and is shown as percentage of GAPDH band intensity. (C) Western blotting of p85α in CU110 cells stably transfected with either lentiviral‐mediated *shPIK3R1* or *SCR*. GAPDH was used as a loading control. Quantitation of western blots is shown on the right. (D) qRT‐PCR results of E‐cadherin, vimentin and EMT‐related transcription factors in CU110 cells stably transfected with either *shPIK3R1* or *SCR* lentivirus. *n *= 3; error bars indicate SD. The results are presented as mean of two different experiments with SD (error bar). Each experiment was done in triplicates. (E) Western blotting of CU110 cells stably transfected with *shAKT1*, *shAKT2* or* SCR*. GAPDH was used as a loading control. Quantitation of western blots is shown in right. (F) qRT‐PCR results of *E‐cadherin, vimentin* and EMT‐related transcription factors in CU110 cells stably transfected with *shAKT1, shAKT2* or *SCR* lentivirus. *n *= 3; error bars indicate SD. The results are presented as mean of two different experiments with SD (error bar). Each experiment was done in triplicate.

**Table S1.** List of shRNA used in the study.
**Table S2.** Sequences of primers used for qPCR analysis.
**Table S3.** Information of HNSCC cell lines, p110α level and sphere‐forming ability.
**Table S4.** Information of various inhibitors used in the screening and their major targets.

## References

[mol212584-bib-0001] Arasada RR , Amann JM , Rahman MA , Huppert SS and Carbone DP (2014) EGFR blockade enriches for lung cancer stem‐like cells through Notch3‐dependent signaling. Cancer Res 74, 5572–5584. 25125655 10.1158/0008-5472.CAN-13-3724 PMC4263272

[mol212584-bib-0002] Argiris A , Karamouzis MV , Raben D and Ferris RL (2008) Head and neck cancer. Lancet 371, 1695–1709. 18486742 10.1016/S0140-6736(08)60728-X PMC7720415

[mol212584-bib-0003] Batlle E and Clevers H (2017) Cancer stem cells revisited. Nat Med 23, 1124–1134. 28985214 10.1038/nm.4409

[mol212584-bib-0004] Bhola NE , Jansen VM , Koch JP , Li H , Formisano L , Williams JA , Grandis JR and Arteaga CL (2016) Treatment of triple‐negative breast cancer with TORC1/2 inhibitors sustains a drug‐resistant and notch‐dependent cancer stem cell population. Cancer Res 76, 440–452. 26676751 10.1158/0008-5472.CAN-15-1640-T PMC4715956

[mol212584-bib-0005] Bornstein S , White R , Malkoski S , Oka M , Han G , Cleaver T , Reh D , Andersen P , Gross N , Olson S *et al* (2009) Smad4 loss in mice causes spontaneous head and neck cancer with increased genomic instability and inflammation. J Clin Invest 119, 3408–3419. 19841536 10.1172/JCI38854 PMC2769185

[mol212584-bib-0006] Brabletz T (2012a) EMT and MET in metastasis: where are the cancer stem cells? Cancer Cell 22, 699–701. 23238008 10.1016/j.ccr.2012.11.009

[mol212584-bib-0007] Brabletz T (2012b) To differentiate or not – routes towards metastasis. Nat Rev Cancer 12, 425–436. 22576165 10.1038/nrc3265

[mol212584-bib-0008] Brabletz T , Kalluri R , Nieto MA and Weinberg RA (2018) EMT in cancer. Nat Rev Cancer 18, 128–134. 29326430 10.1038/nrc.2017.118

[mol212584-bib-0009] Brenner JC , Graham MP , Kumar B , Saunders LM , Kupfer R , Lyons RH , Bradford CR and Carey TE (2010) Genotyping of 73 UM‐SCC head and neck squamous cell carcinoma cell lines. Head Neck 32, 417–426. 19760794 10.1002/hed.21198 PMC3292176

[mol212584-bib-0010] Brown JA , Yonekubo Y , Hanson N , Sastre‐Perona A , Basin A , Rytlewski JA , Dolgalev I , Meehan S , Tsirigos A , Beronja S *et al* (2017) TGF‐beta‐induced quiescence mediates chemoresistance of tumor‐propagating cells in squamous cell carcinoma. Cell Stem Cell 21, 650–664. e658. 29100014 10.1016/j.stem.2017.10.001 PMC5778452

[mol212584-bib-0011] Cancer Genome Atlas Network (2015) Comprehensive genomic characterization of head and neck squamous cell carcinomas. Nature 517, 576–582. 25631445 10.1038/nature14129 PMC4311405

[mol212584-bib-0012] Chen C , Wei Y , Hummel M , Hoffmann TK , Gross M , Kaufmann AM and Albers AE (2011a) Evidence for epithelial‐mesenchymal transition in cancer stem cells of head and neck squamous cell carcinoma. PLoS ONE 6, e16466. 21304586 10.1371/journal.pone.0016466 PMC3029362

[mol212584-bib-0013] Chen D , Wu M , Li Y , Chang I , Yuan Q , Ekimyan‐Salvo M , Deng P , Yu B , Yu Y , Dong J *et al* (2017) Targeting BMI1^+^ cancer stem cells overcomes chemoresistance and inhibits metastases in squamous cell carcinoma. Cell Stem Cell 20, 621–634. e626. 28285905 10.1016/j.stem.2017.02.003 PMC5419860

[mol212584-bib-0014] Chen H , Zhou L , Wan G , Dou T and Tian J . (2011b) BMI1 promotes the progression of laryngeal squamous cell carcinoma. Oral Oncol 47, 472–481. 21482478 10.1016/j.oraloncology.2011.03.016

[mol212584-bib-0015] Cheng H , Liu P , Ohlson C , Xu E , Symonds L , Isabella A , Muller WJ , Lin NU , Krop IE , Roberts TM *et al* (2016) PIK3CA(H1047R)‐ and Her2‐initiated mammary tumors escape PI3K dependency by compensatory activation of MEK‐ERK signaling. Oncogene 35, 2961–2970. 26640141 10.1038/onc.2015.377 PMC4896860

[mol212584-bib-0016] Clay MR , Tabor M , Owen JH , Carey TE , Bradford CR , Wolf GT , Wicha MS and Prince ME (2010) Single‐marker identification of head and neck squamous cell carcinoma cancer stem cells with aldehyde dehydrogenase. Head Neck 32, 1195–1201. 20073073 10.1002/hed.21315 PMC2991066

[mol212584-bib-0017] De Craene B and Berx G (2013) Regulatory networks defining EMT during cancer initiation and progression. Nat Rev Cancer 13, 97–110. 23344542 10.1038/nrc3447

[mol212584-bib-0018] Douma S , Van Laar T , Zevenhoven J , Meuwissen R , Van Garderen E and Peeper DS (2004) Suppression of anoikis and induction of metastasis by the neurotrophic receptor TrkB. Nature 430, 1034–1039. 15329723 10.1038/nature02765

[mol212584-bib-0019] Du L , Chen X , Cao Y , Lu L , Zhang F , Bornstein S , Li Y , Owens P , Malkoski S , Said S *et al* (2016) Overexpression of PIK3CA in murine head and neck epithelium drives tumor invasion and metastasis through PDK1 and enhanced TGFbeta signaling. Oncogene 35, 4641–4652. 26876212 10.1038/onc.2016.1 PMC4985507

[mol212584-bib-0020] Du L , Shen J , Weems A and Lu SL (2012) Role of phosphatidylinositol‐3‐kinase pathway in head and neck squamous cell carcinoma. J Oncol 2012, 450179. 22666248 10.1155/2012/450179 PMC3362130

[mol212584-bib-0021] Fischer KR , Durrans A , Lee S , Sheng J , Li F , Wong ST , Choi H , El Rayes T , Ryu S , Troeger J *et al* (2015) Epithelial‐to‐mesenchymal transition is not required for lung metastasis but contributes to chemoresistance. Nature 527, 472–476. 26560033 10.1038/nature15748 PMC4662610

[mol212584-bib-0022] Gupta PB , Pastushenko I , Skibinski A , Blanpain C and Kuperwasser C (2019) Phenotypic plasticity: driver of cancer initiation, progression, and therapy resistance. Cell Stem Cell 24, 65–78. 30554963 10.1016/j.stem.2018.11.011 PMC7297507

[mol212584-bib-0023] Haddad RI and Shin DM (2008) Recent advances in head and neck cancer. N Engl J Med 359, 1143–1154. 18784104 10.1056/NEJMra0707975

[mol212584-bib-0024] Hanker AB , Kaklamani V and Arteaga CL (2019) Challenges for the clinical development of PI3K inhibitors: strategies to improve their impact in solid tumors. Cancer Discov 9, 482–491. 30867161 10.1158/2159-8290.CD-18-1175 PMC6445714

[mol212584-bib-0025] Hennessy BT , Gonzalez‐Angulo AM , Stemke‐Hale K , Gilcrease MZ , Krishnamurthy S , Lee JS , Fridlyand J , Sahin A , Agarwal R , Joy C *et al* (2009) Characterization of a naturally occurring breast cancer subset enriched in epithelial‐to‐mesenchymal transition and stem cell characteristics. Cancer Res 69, 4116–4124. 19435916 10.1158/0008-5472.CAN-08-3441 PMC2737191

[mol212584-bib-0026] Iglesias‐Bartolome R , Martin D and Gutkind JS (2013) Exploiting the head and neck cancer oncogenome: widespread PI3K‐mTOR pathway alterations and novel molecular targets. Cancer Discov 3, 722–725. 23847349 10.1158/2159-8290.CD-13-0239 PMC4348071

[mol212584-bib-0027] Iliopoulos D , Polytarchou C , Hatziapostolou M , Kottakis F , Maroulakou IG , Struhl K and Tsichlis PN (2009) MicroRNAs differentially regulated by Akt isoforms control EMT and stem cell renewal in cancer cells. Sci Signal 2, ra62. 19825827 10.1126/scisignal.2000356 PMC2862216

[mol212584-bib-0028] Janku F , Yap TA and Meric‐Bernstam F (2018) Targeting the PI3K pathway in cancer: are we making headway? Nat Rev Clin Oncol 15, 273–291. 29508857 10.1038/nrclinonc.2018.28

[mol212584-bib-0029] Kerk SA , Finkel KA , Pearson AT , Warner KA , Zhang Z , Nor F , Wagner VP , Vargas PA , Wicha MS , Hurt EM *et al* (2017) 5T4‐targeted therapy ablates cancer stem cells and prevents recurrence of head and neck squamous cell carcinoma. Clin Cancer Res 23, 2516–2527. 27780858 10.1158/1078-0432.CCR-16-1834 PMC5405006

[mol212584-bib-0030] Koren S , Reavie L , Couto JP , De Silva D , Stadler MB , Roloff T , Britschgi A , Eichlisberger T , Kohler H , Aina O *et al* (2015) PIK3CA(H1047R) induces multipotency and multi‐lineage mammary tumours. Nature 525, 114–118. 26266975 10.1038/nature14669

[mol212584-bib-0031] Kupferman ME , Jiffar T , El‐Naggar A , Yilmaz T , Zhou G , Xie T , Feng L , Wang J , Holsinger FC , Yu D *et al* (2010) TrkB induces EMT and has a key role in invasion of head and neck squamous cell carcinoma. Oncogene 29, 2047–2059. 20101235 10.1038/onc.2009.486 PMC3138334

[mol212584-bib-0032] Lawn S , Krishna N , Pisklakova A , Qu X , Fenstermacher DA , Fournier M , Vrionis FD , Tran N , Chan JA , Kenchappa RS *et al* (2015) Neurotrophin signaling via TrkB and TrkC receptors promotes the growth of brain tumor‐initiating cells. J Biol Chem 290, 3814–3824. 25538243 10.1074/jbc.M114.599373 PMC4319045

[mol212584-bib-0033] Lee SH , Oh SY , Do SI , Lee HJ , Kang HJ , Rho YS , Bae WJ and Lim YC (2014) SOX2 regulates self‐renewal and tumorigenicity of stem‐like cells of head and neck squamous cell carcinoma. Br J Cancer 111, 2122–2130. 25321191 10.1038/bjc.2014.528 PMC4260038

[mol212584-bib-0034] Leemans CR , Braakhuis BJ and Brakenhoff RH (2011) The molecular biology of head and neck cancer. Nat Rev Cancer 11, 9–22. 21160525 10.1038/nrc2982

[mol212584-bib-0035] Levina V , Marrangoni A , Wang T , Parikh S , Su Y , Herberman R , Lokshin A and Gorelik E (2010) Elimination of human lung cancer stem cells through targeting of the stem cell factor‐c‐kit autocrine signaling loop. Cancer Res 70, 338–346. 20028869 10.1158/0008-5472.CAN-09-1102 PMC4572892

[mol212584-bib-0036] Lu H , Clauser KR , Tam WL , Frose J , Ye X , Eaton EN , Reinhardt F , Donnenberg VS , Bhargava R , Carr SA *et al* (2014) A breast cancer stem cell niche supported by juxtacrine signalling from monocytes and macrophages. Nat Cell Biol 16, 1105–1117. 25266422 10.1038/ncb3041 PMC4296514

[mol212584-bib-0037] Lu SL , Herrington H , Reh D , Weber S , Bornstein S , Wang D , Li AG , Tang CF , Siddiqui Y , Nord J *et al* (2006) Loss of transforming growth factor‐beta type II receptor promotes metastatic head‐and‐neck squamous cell carcinoma. Genes Dev 20, 1331–1342. 16702406 10.1101/gad.1413306 PMC1472907

[mol212584-bib-0038] Lui VW , Hedberg ML , Li H , Vangara BS , Pendleton K , Zeng Y , Lu Y , Zhang Q , Du Y , Gilbert BR *et al* (2013) Frequent mutation of the PI3K pathway in head and neck cancer defines predictive biomarkers. Cancer Discov 3, 761–769. 23619167 10.1158/2159-8290.CD-13-0103 PMC3710532

[mol212584-bib-0039] Madsen RR , Knox RG , Pearce W , Lopez S , Mahler‐Araujo B , McGranahan N , Vanhaesebroeck B and Semple RK (2019) Oncogenic PIK3CA promotes cellular stemness in an allele dose‐dependent manner. Proc Natl Acad Sci USA 116, 8380–8389. 30948643 10.1073/pnas.1821093116 PMC6486754

[mol212584-bib-0040] Makino T , Yamasaki M , Takeno A , Shirakawa M , Miyata H , Takiguchi S , Nakajima K , Fujiwara Y , Nishida T , Matsuura N *et al* (2009) Cytokeratins 18 and 8 are poor prognostic markers in patients with squamous cell carcinoma of the oesophagus. Br J Cancer 101, 1298–1306. 19755983 10.1038/sj.bjc.6605313 PMC2768453

[mol212584-bib-0041] Mani SA , Guo W , Liao MJ , Eaton EN , Ayyanan A , Zhou AY , Brooks M , Reinhard F , Zhang CC , Shipitsin M *et al* (2008) The epithelial‐mesenchymal transition generates cells with properties of stem cells. Cell 133, 704–715. 18485877 10.1016/j.cell.2008.03.027 PMC2728032

[mol212584-bib-0042] Maxwell JH , Grandis JR and Ferris RL (2016) HPV‐Associated head and neck cancer: unique features of epidemiology and clinical management. Annu Rev Med 67, 91–101. 26332002 10.1146/annurev-med-051914-021907 PMC5242186

[mol212584-bib-0043] Miettinen M (1991) Keratin subsets in spindle cell sarcomas. Keratins are widespread but synovial sarcoma contains a distinctive keratin polypeptide pattern and desmoplakins. Am J Pathol 138, 505–513. 1704194 PMC1886183

[mol212584-bib-0044] Murai KK and Pasquale EB (2010) Restraining stem cell niche plasticity: a new talent of Eph receptors. Cell Stem Cell 7, 647–648. 21112558 10.1016/j.stem.2010.11.023 PMC3040214

[mol212584-bib-0045] Nassar D and Blanpain C (2016) Cancer stem cells: basic concepts and therapeutic implications. Annu Rev Pathol 11, 47–76. 27193450 10.1146/annurev-pathol-012615-044438

[mol212584-bib-0046] Nguyen LV , Vanner R , Dirks P and Eaves CJ (2012) Cancer stem cells: an evolving concept. Nat Rev Cancer 12, 133–143. 22237392 10.1038/nrc3184

[mol212584-bib-0047] Nieto MA (2011) The ins and outs of the epithelial to mesenchymal transition in health and disease. Annu Rev Cell Dev Biol 27, 347–376. 21740232 10.1146/annurev-cellbio-092910-154036

[mol212584-bib-0048] Nieto MA (2013) Epithelial plasticity: a common theme in embryonic and cancer cells. Science 342, 1234850. 24202173 10.1126/science.1234850

[mol212584-bib-0049] Nor C , Zhang Z , Warner KA , Bernardi L , Visioli F , Helman JI , Roesler R and Nor JE (2014) Cisplatin induces Bmi‐1 and enhances the stem cell fraction in head and neck cancer. Neoplasia 16, 137–146. 24709421 10.1593/neo.131744 PMC3978394

[mol212584-bib-0050] O'Hare T , Shakespeare WC , Zhu X , Eide CA , Rivera VM , Wang F , Adrian LT , Zhou T , Huang WS , Xu Q *et al* (2009) AP24534, a pan‐BCR‐ABL inhibitor for chronic myeloid leukemia, potently inhibits the T315I mutant and overcomes mutation‐based resistance. Cancer Cell 16, 401–412. 19878872 10.1016/j.ccr.2009.09.028 PMC2804470

[mol212584-bib-0051] Oweida A , Bhatia S , Hirsch K , Calame D , Griego A , Keysar S , Pitts T , Sharma J , Eckhardt G , Jimeno A *et al* (2017) Ephrin‐B2 overexpression predicts for poor prognosis and response to therapy in solid tumors. Mol Carcinog 56, 1189–1196. 27649287 10.1002/mc.22574 PMC5939940

[mol212584-bib-0052] Pascual G , Avgustinova A , Mejetta S , Martin M , Castellanos A , Attolini CS , Berenguer A , Prats N , Toll A , Hueto JA *et al* (2017) Targeting metastasis‐initiating cells through the fatty acid receptor CD36. Nature 541, 41–45. 27974793 10.1038/nature20791

[mol212584-bib-0053] Pasquale EB (2010) Eph receptors and ephrins in cancer: bidirectional signalling and beyond. Nat Rev Cancer 10, 165–180. 20179713 10.1038/nrc2806 PMC2921274

[mol212584-bib-0054] Pickering CR , Zhang J , Yoo SY , Bengtsson L , Moorthy S , Neskey DM , Zhao M , Ortega Alves MV , Chang K , Drummond J *et al* (2013) Integrative genomic characterization of oral squamous cell carcinoma identifies frequent somatic drivers. Cancer Discov 3, 770–781. 23619168 10.1158/2159-8290.CD-12-0537 PMC3858325

[mol212584-bib-0055] Poch Martell M , Sibai H , Deotare U and Lipton JH (2016) Ponatinib in the therapy of chronic myeloid leukemia. Expert Rev Hematol 9, 923–932. 27590270 10.1080/17474086.2016.1232163

[mol212584-bib-0056] Prince ME , Sivanandan R , Kaczorowski A , Wolf GT , Kaplan MJ , Dalerba P , Weissman IL , Clarke MF and Ailles LE (2007) Identification of a subpopulation of cells with cancer stem cell properties in head and neck squamous cell carcinoma. Proc Natl Acad Sci USA 104, 973–978. 17210912 10.1073/pnas.0610117104 PMC1783424

[mol212584-bib-0057] Pulte D and Brenner H (2010) Changes in survival in head and neck cancers in the late 20th and early 21st century: a period analysis. Oncologist 15, 994–1001. 20798198 10.1634/theoncologist.2009-0289 PMC3228039

[mol212584-bib-0058] Qiu W , Schonleben F , Li X , Ho DJ , Close LG , Manolidis S , Bennett BP and Su GH (2006) PIK3CA mutations in head and neck squamous cell carcinoma. Clin Cancer Res 12, 1441–1446. 16533766 10.1158/1078-0432.CCR-05-2173 PMC1780023

[mol212584-bib-0059] Saygin C , Matei D , Majeti R , Reizes O and Lathia JD (2019) Targeting cancer stemness in the clinic: from hype to hope. Cell Stem Cell 24, 25–40. 30595497 10.1016/j.stem.2018.11.017

[mol212584-bib-0060] Shien K , Toyooka S , Yamamoto H , Soh J , Jida M , Thu KL , Hashida S , Maki Y , Ichihara E , Asano H *et al* (2013) Acquired resistance to EGFR inhibitors is associated with a manifestation of stem cell‐like properties in cancer cells. Cancer Res 73, 3051–3061. 23542356 10.1158/0008-5472.CAN-12-4136 PMC4506773

[mol212584-bib-0061] Siegel RL , Miller KD and Jemal A . (2017). Cancer Statistics, 2017. CA Cancer J Clin 67, 7–30. 28055103 10.3322/caac.21387

[mol212584-bib-0062] Tabor MH , Clay MR , Owen JH , Bradford CR , Carey TE , Wolf GT and Prince ME (2011) Head and neck cancer stem cells: the side population. Laryngoscope 121, 527–533. 21344428 10.1002/lary.21032 PMC4102680

[mol212584-bib-0063] Thorpe LM , Yuzugullu H and Zhao JJ (2015) PI3K in cancer: divergent roles of isoforms, modes of activation and therapeutic targeting. Nat Rev Cancer 15, 7–24. 25533673 10.1038/nrc3860 PMC4384662

[mol212584-bib-0064] Todoroki K , Ogasawara S , Akiba J , Nakayama M , Naito Y , Seki N , Kusukawa J and Yano H (2016) CD44v3+/CD24‐ cells possess cancer stem cell‐like properties in human oral squamous cell carcinoma. Int J Oncol 48, 99–109. 26647656 10.3892/ijo.2015.3261 PMC4734600

[mol212584-bib-0065] Torre LA , Bray F , Siegel RL , Ferlay J , Lortet‐Tieulent J and Jemal A . (2015). Global cancer statistics, 2012. CA Cancer J Clin 65, 87–108. 25651787 10.3322/caac.21262

[mol212584-bib-0066] Vaishnavi A , Le AT and Doebele RC (2015) TRKing down an old oncogene in a new era of targeted therapy. Cancer Discov 5, 25–34. 25527197 10.1158/2159-8290.CD-14-0765 PMC4293234

[mol212584-bib-0067] Van Keymeulen A , Lee MY , Ousset M , Brohee S , Rorive S , Giraddi RR , Wuidart A , Bouvencourt G , Dubois C , Salmon I *et al* (2015) Reactivation of multipotency by oncogenic PIK3CA induces breast tumour heterogeneity. Nature 525, 119–123. 26266985 10.1038/nature14665

[mol212584-bib-0068] Vander Broek R , Mohan S , Eytan DF , Chen Z and Van Waes C (2015) The PI3K/Akt/mTOR axis in head and neck cancer: functions, aberrations, cross‐talk, and therapies. Oral Dis 21, 815–825. 24219320 10.1111/odi.12206

[mol212584-bib-0069] Zheng X , Carstens JL , Kim J , Scheible M , Kaye J , Sugimoto H , Wu CC , LeBleu VS and Kalluri R (2015) Epithelial‐to‐mesenchymal transition is dispensable for metastasis but induces chemoresistance in pancreatic cancer. Nature 527, 525–530. 26560028 10.1038/nature16064 PMC4849281

[mol212584-bib-0070] Zidar N , Bostjancic E , Malgaj M , Gale N , Dovsak T and Didanovic V (2018) The role of epithelial‐mesenchymal transition in squamous cell carcinoma of the oral cavity. Virchows Arch 472, 237–245. 28699108 10.1007/s00428-017-2192-1

