# Supplementary material for: Distinct roles of PIK3CA in the enrichment and maintenance of cancer stem cells in head and neck squamous cell carcinoma
Source: Mol Oncol. 2019 Oct 26;14(1):139–58. doi: 10.1002/1878-0261.12584 (PMC6944113; doi:10.1002/1878-0261.12584)
Supplement: Supplementary file 1 — Fig. S1. Overexpression of PIK3CA promotes EMT and de‐differentiation. (A) Immunofluorescence staining of E‐cadherin (green) and vimentin (red) in two CUCON and two CU110 cell lines. DAPI stain (blue) was used to visualize the nucleus. Scale bar: 50 µm. (B) qRT‐PCR analysis of cytokeratins in CUCON and CU110 cell lines. The results are presented as mean of two different experiments with standard deviations (error bar). Each experiment was done in triplicate. *P < 0.05. (C) Immunohistochemistry staining of CD44 (left), SOX2 (middle), and BMI1 (right) in the 4NQO‐induced tumors from either the PIK3CA or control mice; n = 3 for each group. SOX2+ cells were enumerated in three independent fields from three PIK3CA‐tumors and three control‐tumors. The quantitation result is included in the text. Scale bar: 50 µm (CD44 and BMI1), 25 µm (SOX2). Fig. S2. qRT‐PCR analysis of ALDH1A1 and ABCG2 in CU110 cells cultured in either monolayer or sphere. The results are presented as mean of two different experiments with standard deviations (error bar); Each experiment was done in triplicate. *P < 0.05. Fig. S3. Knocking down of PIK3CA failed to reverse EMT. Immunofluorescence staining of E‐cadherin or vimentin in CU110‐2 cells stably transfected with either shPIK3CA or a scrambled control (SCR). DAPI staining (blue) was used to visualize the nucleus. Scale bar: 50 µm. Fig. S4. Effect of knocking down of PIK3CA on human head and neck cancer cell lines. (A) PIK3CA expression in 17 human head and neck cancer cell lines (as indicated) using qRT‐PCR. Experiment was done in triplicate. (B) Western blotting of p110α, AKT, pAKT(Ser473) and GAPDH in Fadu (left) and UMSCC47 cell lines (right) stably transfected with either lentiviral‐mediated shPIK3CA or SCR. Quantitation of western blots is shown on the right. (C) Cell proliferation assay of Fadu (left) and UMSCC47 (right) cell lines stably transfected with either shPIK3CA or SCR. n = 3; error bars indicate SD. *P < 0.05. Fig. S5. Examinatio [file MOL2-14-139-s001.pptx]

## Slide 1
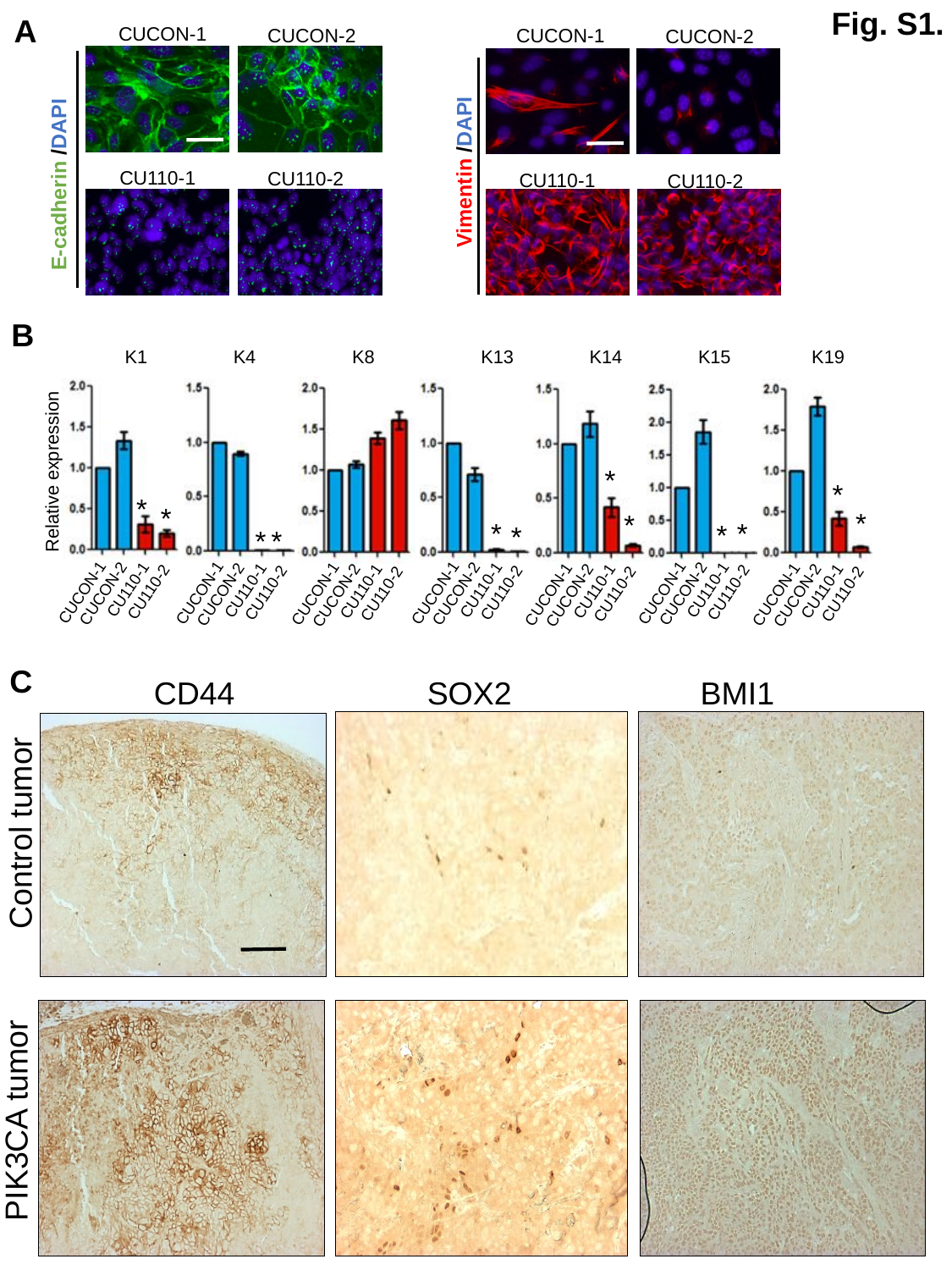

Fig. S1.
A
CUCON-1
CUCON-1
CUCON-2
 Vimentin /DAPI
CU110-1
CU110-2
CUCON-2
E-cadherin /DAPI
CU110-1
CU110-2
B
 K1 K4 K8 K13 K14 K15 K19
Relative expression
CU110-1
CU110-2
CUCON-1
CUCON-2
CU110-1
CU110-2
CUCON-1
CUCON-2
CU110-1
CU110-2
CUCON-1
CUCON-2
CU110-1
CU110-2
CUCON-1
CUCON-2
CU110-1
CU110-2
CUCON-1
CUCON-2
CU110-1
CU110-2
CUCON-1
CUCON-2
CU110-1
CU110-2
CUCON-1
CUCON-2
*
*
*
*
*
*
*
*
*
*
*
*
C
BMI1
CD44
SOX2
Control tumor
PIK3CA tumor

## Slide 2
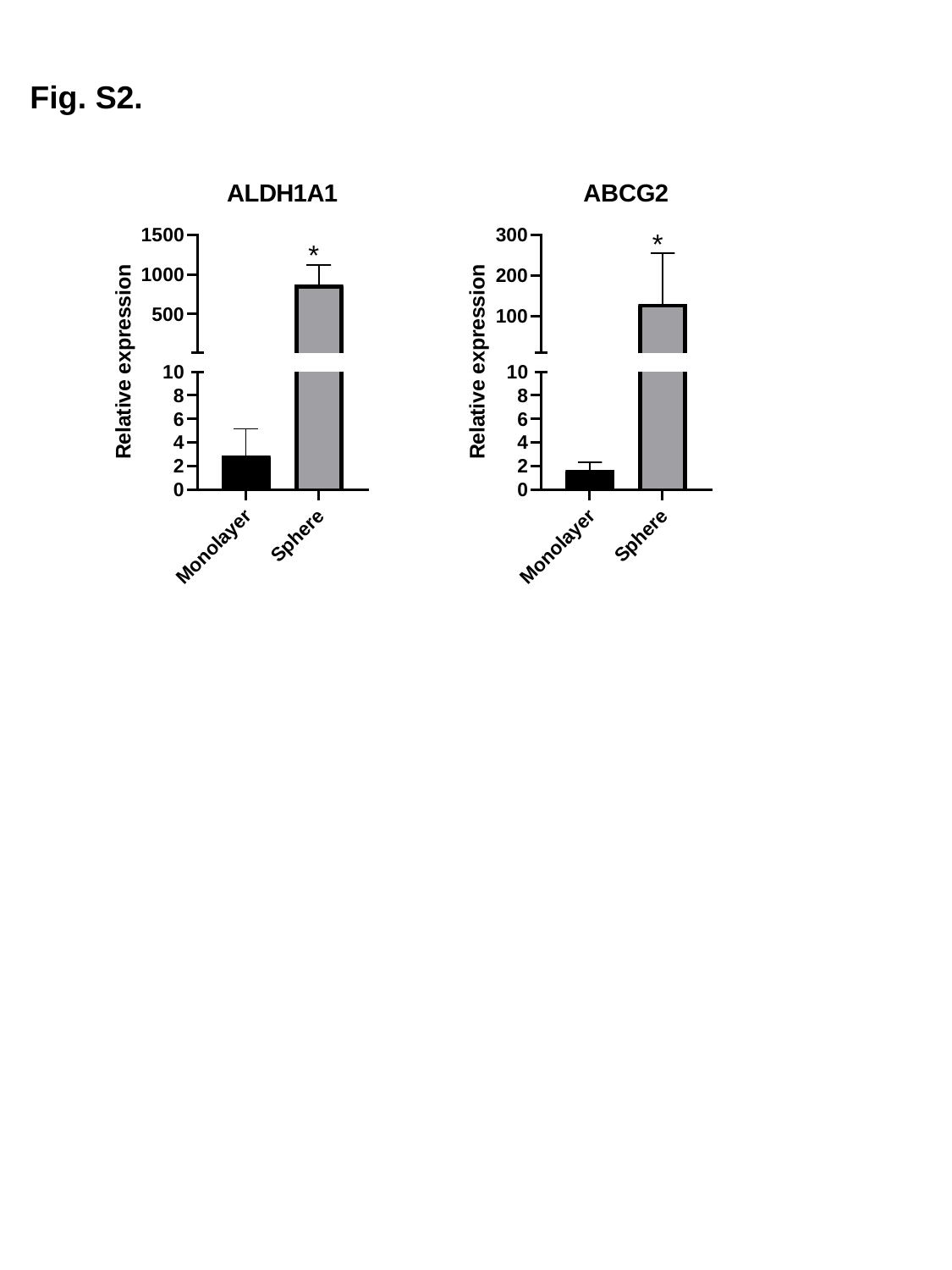

Fig. S2.
*
*

## Slide 3
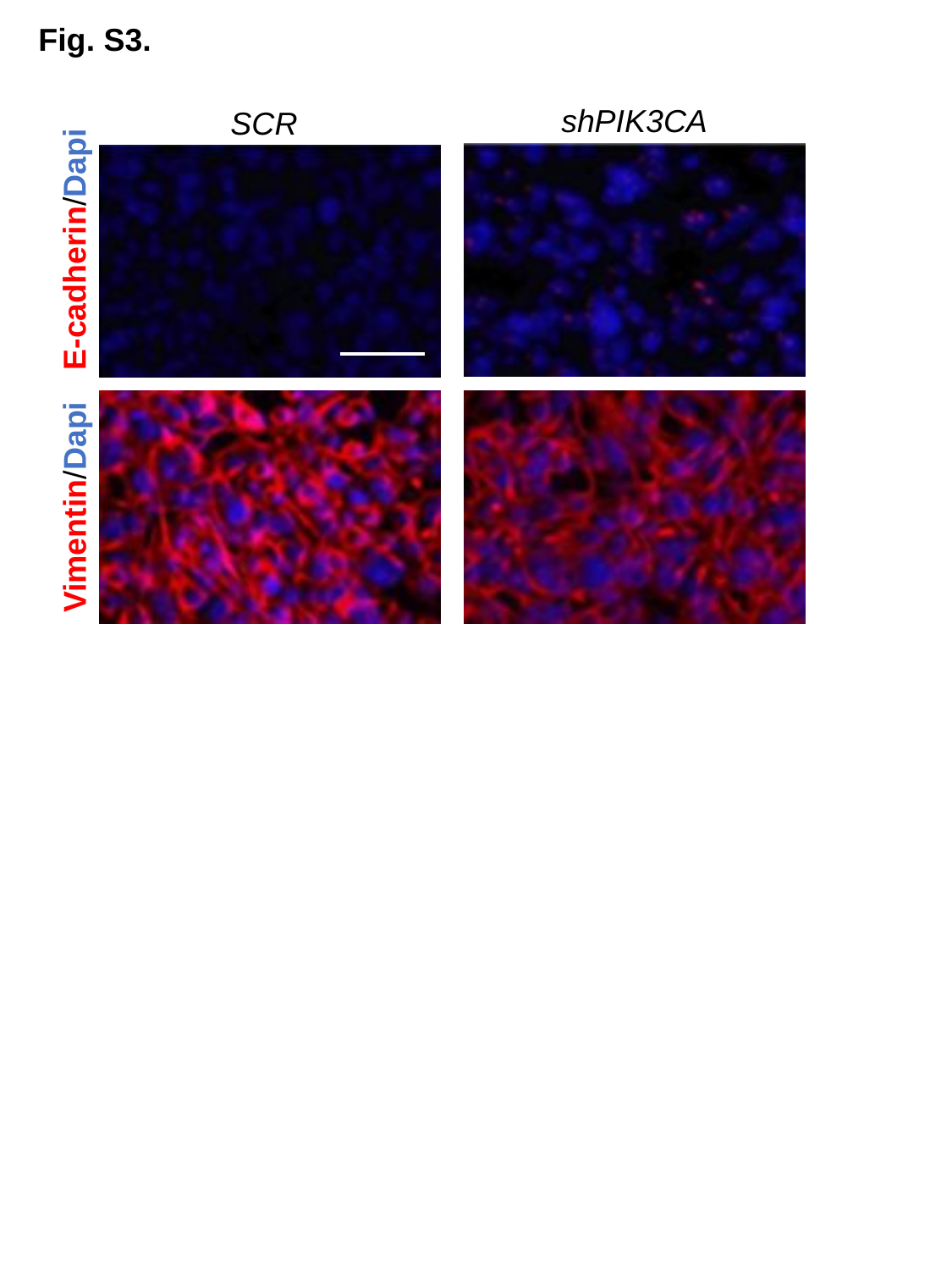

Fig. S3.
shPIK3CA
SCR
E-cadherin/Dapi
Vimentin/Dapi

## Slide 4
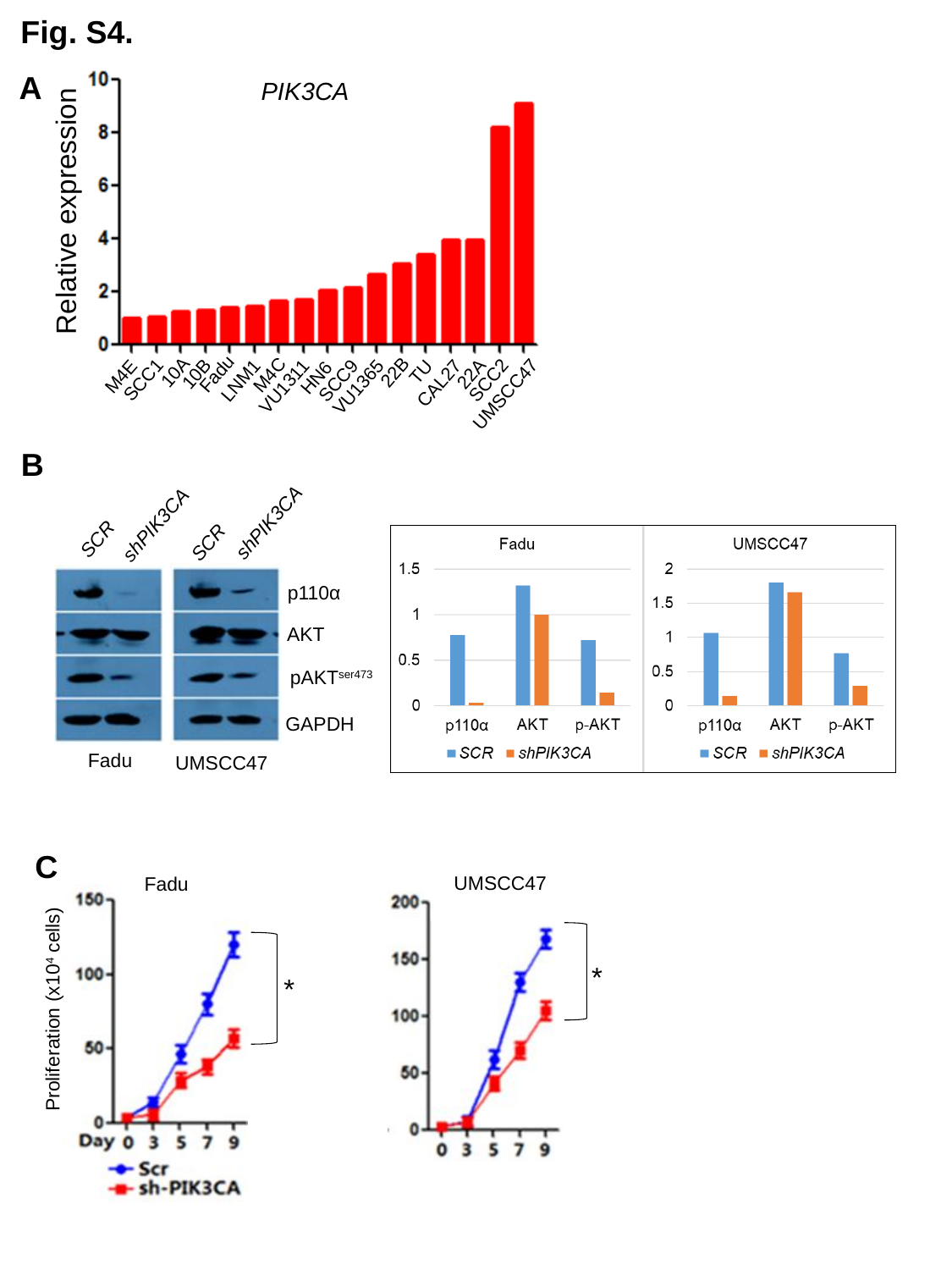

Fig. S4.
PIK3CA
Relative expression
10A
TU
22B
10B
22A
Fadu
M4C
M4E
HN6
SCC1
SCC2
SCC9
LNM1
CAL27
VU1311
VU1365
UMSCC47
A
B
shPIK3CA
shPIK3CA
SCR
SCR
p110α
AKT
pAKTser473
GAPDH
Fadu
UMSCC47
C
UMSCC47
Fadu
*
*
Proliferation (x104 cells)

## Slide 5
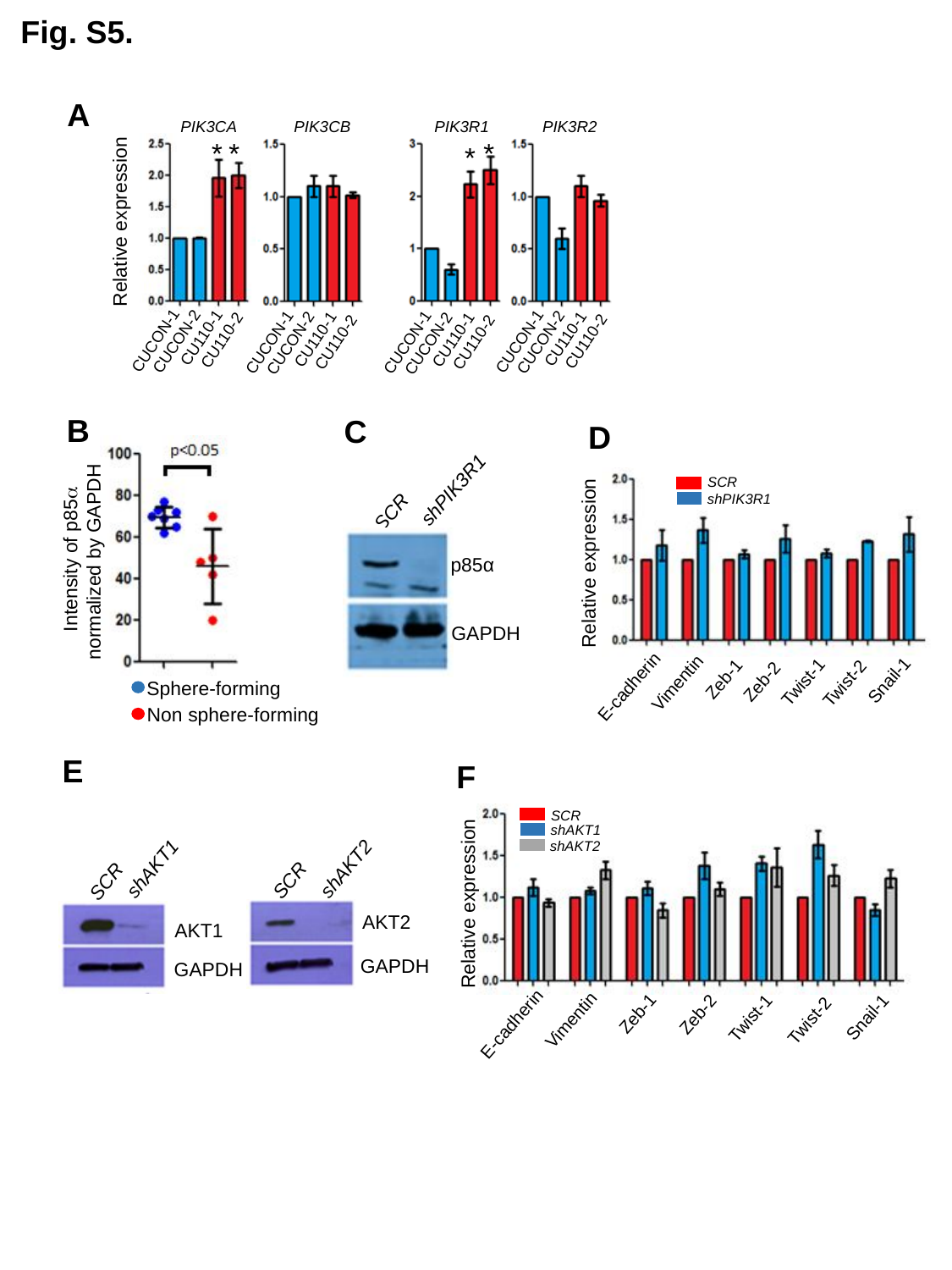

Fig. S5.
A
PIK3CA PIK3CB 	PIK3R1 PIK3R2
*
*
*
*
Relative expression
CU110-1
CU110-2
CUCON-1
CUCON-2
CU110-1
CU110-2
CUCON-1
CUCON-2
CU110-1
CU110-2
CUCON-1
CUCON-2
CU110-1
CU110-2
CUCON-1
CUCON-2
B
C
D
Intensity of p85a normalized by GAPDH
Sphere-forming
Non sphere-forming
Relative expression
Zeb-1
Snail-1
Zeb-2
Twist-1
Vimentin
Twist-2
E-cadherin
SCR
shPIK3R1
shPIK3R1
SCR
p85α
GAPDH
E
F
SCR
shAKT1
shAKT2
Relative expression
Zeb-1
Zeb-2
Snail-1
Twist-1
Vimentin
Twist-2
E-cadherin
shAKT1
SCR
AKT1
GAPDH
shAKT2
SCR
AKT2
GAPDH
