# Supplementary material for: Distinct roles of PIK3CA in the enrichment and maintenance of cancer stem cells in head and neck squamous cell carcinoma
Source: Mol Oncol. 2019 Oct 26;14(1):139–58. doi: 10.1002/1878-0261.12584 (PMC6944113; doi:10.1002/1878-0261.12584)
Supplement: Supplementary file 2 — Table S1. List of shRNA used in the study. Table S2. Sequences of primers used for qPCR analysis. Table S3. Information of HNSCC cell lines, p110α level and sphere‐forming ability. Table S4. Information of various inhibitors used in the screening and their major targets. [file MOL2-14-139-s002.docx]

**Supplementary Tables.**

**Table S1. List of shRNAs used in the study.**

| sh-RNA | TRC number |
| --- | --- |
| sh-PIK3CA (m) | TRCN0000361413 |
| sh-AKT1 (m) | TRCN0000039797 |
| sh-AKT2 (m) | TRCN0000265834 |
| sh-PIK3R1 (m) | TRCN0000302323 |
| sh-PIK3CA (h) | TRCN0000010406 |
| pLKO Non-target shRNA control | SHC216 |
| pLKO Non-target shRNA control | shCntrl-1 |

**Table S2. Sequences of primers used for qPCR analysis.**

| Gene | Species | Sequence (F) | Sequence (R) |
| --- | --- | --- | --- |
| CDH1 | M | 5'-CATGTTCACTGTCAATAGGG | 5'-GTGTATGTAGGGTAACTCTCTC |
| vimentin | M | 5'-GAACCTGAGAGAAACTAACC | 5'-GATGCTGAGAAGTCTCATTG |
| ZEB1 | M | 5'-ATATGAGCACACAGGTAAGAG | 5'-TTCATGTGTTGAGAGTAGGAG |
| ZEB2 | M | 5'-CAAACAAGCCAATCCCAGGAG | 5'-TCCAGAGGGTTTGCAAGGCTA |
| SNAIL1 | M | 5'-AGTTGACTACCGACCTTG | 5'-AAGGTGAACTCCACACAC |
| SNAIL2 | M | 5'-GACACATTAGAACTCACACTG | 5'-GACATTCTGGAGAAGGTTTTG |
| TWIST1 | M | 5'-GAGACCTAGATGTCATTGTTTC | 5'-GAATTTGGTCTCTGCTCTTC |
| TWIST2 | M | 5'-CGCATACTCCTGTTCTTTAC | 5'-CTCTTTATTGTTCCTGGGTG |
| MK1 | M | 5'-ACGCAGAGAATGAGTTCGTGA | 5'-TCAATGTCTTGCTGGAGGGCA |
| MK5 | M | 5'-ACCCTCAACAACAAGTTTGCC | 5'-TCTGCTTTATGGTCTTGGTGC |
| MK8 | M | 5'-AGCTGAGGCTGAAACCATGTA | 5'-TCTGGCCTTTGAGGGCTTCAA |
| MK13 | M | 5'-ATTCAGCAACCAGGTAGTAGG | 5'-TGGTCTGGAACCATTCCTCAG |
| MK14 | M | 5'-CGATGACTTCCGGACCAAGTT | 5'-TGAGGCTCTCAATCTGCATCT |
| MK15 | M | 5'-TCAATGTGGAAATGGACGCAG | 5'-TGTTAGACGCCACCTCCTTGT |
| SOX2 | M | 5'-GCGGAGTGGAAACTTTTGTCC | 5'-CGGGAAGCGTGTACTTATCCTT |
| NANOG | M | 5'-TTGCTTACAAGGGTCTGCTACT | 5'-ACTGGTAGAAGAATCAGGGCT |
| OCT4A | M | 5'-AGTTGGCGTGGAGACTTTGC | 5'-CAGGGCTTTCATGTCCTGG |
| PIK3CA | M | 5'-TGCCTGTGGGATGTATCTGAA | 5'-TGCAAAGCATCCATGAAGTCT |
| PIK3CB | M | 5'-GTCTTGGATCGACTGGCTAAA | 5'-ACAACCAGCTTTCCTCCATAA |
| PIK3CD | M | 5'-ACGAAGTCCAGGAGCATTTC | 5'-CCAGGAGCACAGCAAATAGA |
| PIK3CG | M | 5'-TCCATCCTGCTGGACAATTAC | 5'-CGAAGCTGATTGGGCATTTC |
| PIK3R1 | M | 5'-CTTGGAGATGATCGACGTACAC | 5'-GCTCTGTAGTTCTTGGGCTAAA |
| PIK3R2 | M | 5'-AGGGAAGAGGTGAATGAGAGA | 5'-CGTGTACTCTCCTTGGATCTTG |
| PIK3R5 | M | 5'-CAGGACAGCTTTGACATCCTAC | 5'-CTCCTCTTCCTCCTCTTCATCA |
| PIK3R6 | M | 5'-GGCACTCAGCCCATCTATTT | 5'-GGAGTCTTGGATCTTCACCTTC |
| PIK3C2A | M | 5'-CTGGAAGTGACACAAGGAAGAA | 5'-CATGGAGCCTGAGAAGATCATAA |
| PIK3C3 | M | 5'-GCTCAGCAGACCTTTGTAGAT | 5'-CAAGGCCTGAAGTCTCTCATT |
| GAPDH | M | 5'-AGGTCGGTGTGAACGGATTTG | 5'-TGTAGACCATGTAGTTGAGGTCA |
| PIK3CA | H | 5'-ATTGGCTTCTCAAAGATGCCC | 5'-TTGGAGAACAACCTGTCTGGC |
| PIK3R1 | H | 5'-CAGCAGCCAGCTCTGATAATA | 5'-AGGCTGTCGTTCATTCCATTC |
| ACTB | H | 5'-CGTGACATTAAGGAGAAGCTG | 5'-CTAGAAGCATTTGCGGTGGAC |

**Table S3. Information of HNSCC cell lines, p110α level and sphere forming ability**

| **Cell lines** | **Site, Gender, Age** | **HPV** | **Intensity of p110a normalized by GAPDH)** | **Sphere forming ability** |
| --- | --- | --- | --- | --- |
| UMSCC10B | Lymph node, M,57 | negative | 96.7 | yes |
| UMSCC22B | Lymph nodes, F, 58 | negative | 95.2 | yes |
| UMSCC22A | Hypopharynx, F, 58 | negative | 78.5 | yes |
| VU1365 | Oral cavity, M, 22 | n/a | 64.3 | yes |
| UMSCC47 | Tongue, M | positive | 62.5 | yes |
| Cal27 | Tongue, M, 56 | negative | 61.1 | yes |
| VU1131 | Floor of mouth, 34F | n/a | 58.5 | yes |
| Fadu | Hypopharynx, M, 56 | negative | 55.9 | yes |
| UMSCC1 | Floor of mouth, M, 60 | negative | 48.2 | yes |
| Tu167 | Floor of mouth, M, 72 | negative | 38.8 | yes |
| HN6 | Tongue, M, 54 | negative | 22.6 | yes |
| LNM1 | Lymph node of M4c | n/a | 48.8 | no |
| UMSCC10A | Larynx, M, 57 | negative | 46.9 | no |
| M4E | Base of tongue | n/a | 39.9 | no |
| M4c | Base of tongue | n/a | 38.2 | no |
| UMSCC2 | Oral cavity, F, 64 | negative | 37.1 | no |
| SCC9 | Tongue, M, 25 | negative | 21.7 | no |

**Table S4. Information of various inhibitors used in the screening and their major targets.**

| Inhibitor | Vendor | Cat. No. | Target/s | IC_50_ (µM) |
| --- | --- | --- | --- | --- |
| PX866 | LC laboratories | P-7501 | pan-PI3K | 5.89 |
| gefitinib | Selleck Chemicals | S1025 | EGFR | 9.56 |
| imatinib | Selleck Chemicals | S2475 | c-kit, PDGFR | 33.0 |
| axitinib | Selleck Chemicals | S1005 | VEGFR | 1.80 |
| ponatinib | Selleck Chemicals | S1490 | Multi-RTKs | 0.54 |
| AZD4547 | Selleck Chemicals | S2801 | FGFR | 4.40 |
| BMS-777607 | Selleck Chemicals | S1561 | Axl, Ron, Met, Tyro3 | 7.12 |
| GNF-5837 | Selleck Chemicals | S7519 | TrkA/B/C | 6.15 |
| OSI906 | Selleck Chemicals | S1091 | IGF-1R, InsR | 24.0 |
| Paclitaxel | Selleck Chemicals | S1150 | Microtubule | 0.08 |
| Everolimus | Selleck Chemicals | S1120 | mTOR | 12.92 |
| LDN211904 | Merck Millipore | 428201 | Ephrin receptors | 8.73 |
